# Technological Solutions and Contested Interpretations of Scientific Results: Risk Assessment of Diesel Emissions in the United States and in West Germany, 1977–1995

**DOI:** 10.1007/s00048-020-00276-2

**Published:** 2020-10-07

**Authors:** Christopher Neumaier

**Affiliations:** 1 Fakultät für Geistes- und Sozialwissenschaften, Helmut-Schmidt-Universität Hamburg, Postfach 70 08 22, 22008 Hamburg, Germany; 2 Leibniz-Zentrum für Zeithistorische Forschung Potsdam, Am Neuen Markt 1, 14467 Potsdam, Germany

**Keywords:** Diesel Car, Risk Assessment, Science, Technology, Policy, Germany, USA, Dieselauto, Risikoeinschätzung, Wissenschaft, Technik, Politik, Deutschland, USA

## Abstract

This article traces the different classifications of diesel emissions either as “safe” or as “hazardous” in the US and in West Germany between 1977 and 1995. It argues that the environmental regulation of diesel emissions was a political threshold. It contributes to our general understanding of how politicians, environmental lobbyists, scientists, and engineers constructed the standards and norms that defined the “safe” limit of environmental pollutants. After discussing how diesel emissions came under review as a potential carcinogen, I will show that the coding as “safe” or as “hazardous” resulted from negotiations that were entirely dependent on the temporal, geographical, and intellectual contexts in which diesel technology, scientific research on their emissions, and political regulation were embedded. In particular, I trace the differences in German and US regulatory policy. While US regulation relied more on epidemiology that provided only weak data on the carcinogenicity of diesel particulates in the early 1980s, German government agencies tended to base their policy around the mid-1980s more on the results of animal tests and shortly afterwards also on epidemiology. Furthermore, the article reveals how US and German automakers tried to foster doubt on the carcinogenicity of diesel emissions and how their approaches differed and shifted. Thereby, it sheds light on the triangular relationship between technology, science, and politics in regulatory processes by analyzing the different roles of the state, automakers, scientists, and environmental agencies in Germany and in the United States.

## Abstract (de)

Dieser Beitrag analysiert, wie sich die Einstufung der Dieselabgasemissionen entweder als „gesundheitlich unbedenklich“ oder als „gesundheitsschädlich“ in den USA und in Westdeutschland zwischen 1977 und 1995 wandelte. Es wird argumentiert, dass die eingeführten Abgasgrenzwerte politisch definiert wurden. Damit wird mit diesem Artikel ein Beitrag zu der Frage geleistet, wie Politiker, Umweltschützer, Wissenschaftler und Ingenieure die Abgasstandards und -normen definierten, die als „unbedenklich“ galten. Nachdem eingangs diskutiert wird, warum Dieselabgasemissionen als mögliches Karzinogen galten, zeige ich anschließend, wie das Kodieren der Schadstoffe als „gesundheitlich unbedenklich“ oder als „gesundheitsschädlich“ das Resultat von Verhandlungen war. Deren Ergebnisse waren wiederum abhängig von den spezifischen zeitlichen, geographischen und intellektuellen Kontexten, in die die Dieseltechnik, die wissenschaftliche Forschung zu Abgasemissionen und die politische Regulierung von Schadstoffen eingebettet waren. Insbesondere werden dabei die Unterschiede der deutschen und der US-amerikanischen Regulierungspolitiken herausgearbeitet. Während in den USA politische Entscheidungsträger sich Anfang der 1980er Jahre vor allem auf epidemiologische Daten verließen, die nur ein schwaches krebserregendes Potenzial von Dieselabgasen aufzeigten, basierten deutsche Regierungsbehörden ihr Urteil nach 1985 zunächst vor allem auf den Ergebnissen von Tierversuchen und kurze Zeit später auch auf epidemiologischen Studien. Zudem zeigt der Artikel, wie US-amerikanische und deutsche Automobilhersteller versuchten, Zweifel an der Kanzerogenität von Dieselpartikeln zu streuen, und wie sich ihre Herangehensweisen unterschieden und veränderten. In dem die Rolle des Staates, der Automobilhersteller, der Wissenschaftler und der Umweltbehörden in Deutschland und in den USA in den Blick genommen wird, zeigt dieser Beitrag auf, wie sich die drei Bezugspunkte Technik, Wissenschaft und Politik bei Regulierungsprozessen zueinander verhielten.

## Introduction

In 2018, American and German media outlets reported that scientists had forced monkeys to inhale diesel emissions four years earlier in an attempt to prove their harmlessness. These outlets revealed that the *Europäische Forschungsvereinigung für Umwelt und Gesundheit im Transportsektor* (EUGT) had in fact tasked the Lovelace Respiratory Research Institute (LRRI) in Albuquerque, New Mexico, with this specific animal test. Furthermore, they also unveiled that not only was the EUGT a lobbying group for the German automotive industry under the aegis of Volkswagen, but also that other than its name insinuated it was no research institute. The naming intended to disguise the link to the automobile industry and thus foster within the general public more credible results, rehabilitating diesel cars from their “dirty” image.

This plan flopped when media publicized the ethically problematic test structure with monkeys. Reactions of utter disgust reverberated among politicians, scientists, and the general public. Several of such ethically dubious experiments had been conducted, including one inhalation experiment, which exposed twenty-five humans to nitrogen dioxide emissions (NO_2_). This lays bare a strategy whereby science should help to promote “clean” diesel technology, in particular by liberating diesel cars from the cancer-causing stigma that emanated from their NO_x_ and particulate emissions.^1^ This scandal also sheds light on how Volkswagen found its path to what its engineers referred to as “clean” diesel technology that was “safe” for public health.

This article traces the different classifications of diesel emissions as either “safe” or “hazardous” in the US and in West Germany between 1977 and 1995 as well as the ways in which those designations were not only negotiated, but also ultimately coded. In analyzing scientific and political approaches towards diesel emissions and linking them to engineering principles, I will not only show how the different coding of diesel cars emerged but likewise how environmental regulation were political thresholds. Thereby, I will also expand our general understanding of how politicians, environmental lobbyists, scientists, and engineers constructed the standards and norms that defined the “safe” limit of environmental pollutants between the late 1970s and early 1990s.

In this article, I first discuss when and why diesel particulate emissions came under review as a potential carcinogen in the USA in the late 1970s. This initiated massive research in the United States on the technological and engineering solutions that promised “cleaning” diesel emissions. Moreover, research reviewed how political measures could affect diesel emissions control technology, as shown in the following section. These findings indicate one major argument: the coding of emissions as “safe” or “hazardous” results from malleable negotiations that are entirely dependent on the temporal, geographical, and intellectual contexts into which diesel technology, scientific research on their emissions, and political regulation are embedded. This becomes even more apparent when the failure of diesel particulate filter technology and its detrimental impact on the reputation as well as on the sales of diesel cars in the US is discussed in the subsequent chapter. Consequently, I argue that, in addition to emotions (Neumaier 2020), three other factors had a significant impact on the cultural acceptance of diesel engine technology: the reputation of diesel cars and the technological solutions to “clean-up” diesel emissions; scientific research on the health effects of emissions; and political regulation.

Diesel cars sales plummeted in the US at a time when research had not yet been able to provide conclusive scientific evidence on the carcinogenicity of diesel exhaust. The section on ambiguities shows how this created a window of opportunity for industry-sponsored scientific research. It tried to sow doubt on the cancer-causing effect of diesel exhaust by highlighting potential uncertainties and by presenting contradictory research results—a common approach of industry representatives during the twentieth century (Langston 2010: 44; Oppenheimer et al. 2019: 11–18; Oreskes & Conway 2010: 16; Vogel 2012: 17). In addition, I show in this and the following chapter how US regulation relied on public and adversarial hearings that based their lines of argumentation on formal methods such as epidemiology; risk analysis; and, to a lesser degree, on animal tests provided by scientists. Moreover, political decision making weighed these assessments of threat to public health against the economic impact of regulatory resolutions, as Sheila Jasanoff has demonstrated with her analysis of scientific “risk assessment” and political “risk management” (Jasanoff 1990: 185). Risk assessment dealt with scientific evidence on the health risks of chemical compounds such as ozone and diesel particulates. Risk management also included the estimates on economic consequences of regulatory decisions for the automotive industry. Yet, at the federal level in the US, it further tended to favor economic costs over threats to public health, if scientific evidence was inconclusive (Jasanoff 1990: 110–111, 185; Jasanoff 2005: 17–18; Langston 2010: 26; Neumaier 2014: 438; Oreskes & Conway 2010: 142–143).

Researchers have argued that in the 1980s the precautionary principle (limiting the emission of pollutants even when scientific evidence on their harmfulness had still been lacking) did not guide the regulation of automobiles (Langston 2010: vii–ix; Vogel 2012: 35–36). My sources challenge this assessment in the chapter on cancer risk calculation, indicating that the State of California and its Air Resources Board (CARB) in fact applied the precautionary principle when it came to limiting the emission of airborne chemical compounds, such as diesel particulates in the early 1980s. Since then, regulatory policy in California—and in ten other states that adopted California’s standards—has remained far more rigorous than the US federal level. Furthermore, these regulations also exceeded the initially soft European emission standards that were not tightened until the early twenty-first century (Vogel 2012: 117).

Then, my essay will contrast US regulatory policy with the German approach that can serve as a paradigmatic West European case study. I will show that research on the health risks of chemical substances started later in West Germany than in the US. In addition, the scientific and political methods varied. German studies incorporated both epidemiology and animal tests. However, by the mid-1980s, animal testing represented most scientific evidence on the health threats of diesel particulates emissions. At the end of the decade, epidemiological research also started to indicate strongly the considerable cancer-causing potential of diesel emissions. Yet, I will illustrate that German political regulators not only relied on the scientific evidence on health risks, but also established their own data concerning the dangers, which stemmed from negotiations with automotive industry representatives. They continuously tried to create doubt regarding the carcinogenicity of diesel particulates, in particular by presenting contradicting interpretations and questioning the scientific methods of independent toxicologists. Ultimately, this gave even more leverage to economic interests over established science, which will be discussed in the final chapter.

By reviewing the different roles of the state, automakers, scientists, and environmental agencies in Germany and in the United States, this article examines both, how the public reacted as well as the diverging public perceptions of diesel emissions as either “safe enough” or hazardous that emerged in the 1970s and 1990s. This considerably expands my previous research, which primarily discussed emotionally-charged public debates on diesel cars and their political impacts (Neumaier 2010a; Neumaier 2010b; Neumaier 2010c; Neumaier 2020). The triangular relationship between technology, science, and politics in regulatory processes not only offers new perspectives on the history of diesel cars, but also on the interplay of engineering principles, scientific research, and political decisions.

I base my argument on governmental records, car magazines, and reports published by automakers. Further, I widen the lens of previous scholarship by including new perspectives on the carcinogenicity as well as the risk assessment and risk calculation of diesel exhaust fumes by scientists and engineers. This essay establishes the mechanisms by which available pollution control technology, scientific approaches, and contemporary social contexts caused diverging risk perceptions of diesel emissions that eventually led to two different models of regulatory policy towards environmental pollutants in Europe and the USA.

## Cancer Research, Public Fears, and the Role of Diesel Cars in the USA During the 1970s

Cancer rose to prominence in the American consciousness after President Richard M. Nixon (1913–1994) promised to increase efforts to find a cure for the disease in his January 1971 State of the Union address. Nine months later, he signed the National Cancer Act into law. Although Nixon’s public announcement raised awareness of cancer in the US, it was the enactment of the legislation that provided the financial and institutional backing to initiate nationwide research endeavors such as of the National Cancer Institute. Scientists observed the formation of tumors and investigated possible cures and, in turn, their ongoing research targeted automobile emissions. Scientists tried to assess whether they possessed a carcinogenic potential. In addition, scientific advisory committees formed and provided expert opinions for the nascent political regulation (Jasanoff 1986: 17–24; Jasanoff 1990: 2–3, 43; Neumaier 2014: 440–441; Proctor 1995: 250–257).^2^

In the winter of 1977/78, preliminary reports surfaced that the Environmental Protection Agency (EPA) was studying the carcinogenicity of diesel emissions as the effects of diesel particulate matter—a potentially hazardous chemical compound—had come into question following a study conducted under their purview. An EPA laboratory in Research Triangle Park, North Carolina, had used the Ames test, a standard biological assay, to determine the mutagenicity of chemical compounds. The results were unequivocal: salmonella bacteria mutated once exposed to diesel exhaust fumes. Diesel particulates were thus identified as a mutagen. Since biologists and physicians knew that many mutagens acted as carcinogens, they hinted that diesel particulate emissions could also be cancer-causing.^3^ Yet, they were unable to answer the question on the carcinogenicity of diesel particulates with a definitive “yes.” This remained an important issue because epidemiological data or animal tests only enable researchers to determine indirectly the cancer risks for humans. Hence, some uncertainties always remain, and can be wielded to establish counter-evidence on the non-toxicity of substances, as done with the tobacco industry on other cases (Oreskes & Conway 2010). Despite lacking irrefutable scientific evidence, scientists and politicians reviewed the health risks of diesel emissions. This subsequently sparked controversy within an emotionally-charged American public, which considered diesel particulate emissions a threat to their health around 1980/81 (Neumaier 2020: 88–91). Diesels were put in the spotlight for three reasons.

First, historically both gasoline- and diesel-powered cars gave off particulate emissions. This similarity vanished with the introduction of unleaded gasoline in the US during the mid-1970s. While gasoline-powered cars emitted lead particles in the range of c. 0.25 g/mi, the usage of unleaded gasoline immediately dropped particulates to a negligible average of 0.01 g/mi. Diesels, in contrast, continued to emanate higher averages of 0.60 g/mi. Notably, at 1.0 g/mi, the GM-produced Oldsmobile diesels’ emissions reached the hundred-fold level of their gasoline-powered cousins.^4^

Second, the EPA had in any case been planning to introduce a particulate standard for diesel cars. The agency made this move because the Clear Air Act Amendments of 1977 mandated improving the National Ambient Air Quality Standards by setting standards for substances including ozone (O_3_), sulfur dioxide (SO_2_), lead, and particulate matter. Significantly, it was the framework of the Clean Air Act that triggered political decisions to limit emissions of particulates and not the ongoing cancer speculations. This subtle differentiation, however, was not explicitly addressed in public and is important because it eventually led to a diverging public perception of diesel emissions. While the regulation of diesel particulates triggered cancer fears among US citizens, politicians and scientists remained cautious labelling diesel as “cancer-causing” as long as scientific evidence had not been provided (Jasanoff 1990: 102–106; Jasanoff 1992: 213–214; McCarthy 2007: 178–180; Neumaier 2014: 436–437).

**Fig. 1:**
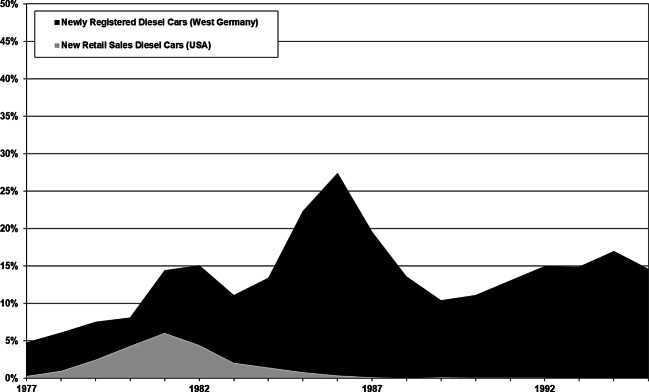
Registration and Retail Sales of Diesel Cars in West Germany and in the USA, 1977–1995. (Source: Davis & Diegel 2004; Kraftfahrt-Bundesamt 1995)

Third, diesel car sales increased rapidly between 1977 and 1981, as shown in Fig. 1, from a share of 0.35 percent of new retail sales to an all-time high of 6.1 percent nationwide, reaching even nine percent in California.^5^ Car enthusiast magazines such as *Motor Trend* proclaimed in 1979 that a “Dieselmania” swept the USA and was about to revolutionize the passenger car market. Several unique factors had initiated the trend towards diesel cars.^6^ For instance, diesel cars offered by Mercedes-Benz, Volkswagen, and General Motors appealed to consumers because of their technological characteristics. Moreover, the US was in the midst of the second energy crisis of the 1970s: the supply of gasoline was limited and led to long lines of cars waiting in front of gas stations in 1979. It further reminded the public not only of the oil shock in 1973, but also about the United States’ vulnerability and its dependence on foreign crude oil. Diesel fuel, in contrast to gasoline, was plentiful and the frugal diesel automobile, with its well-known high gas mileage, appeared a prudent choice. One year later, *Car and Driver *shared this viewpoint and advised its readers that “diesels are here to stay, their numbers are visibly increasing, and yesterday’s weirdness is simply today’s common sense.”^7^

The economic advisers of President Jimmy Carter also predicted strong growth in diesel cars on the road and this created increased political pressure for both the Carter Administration and the EPA to monitor the impact of diesels on public health more closely and to review the economic consequences of the regulation of diesel cars. Indeed, some estimates projected that, by 1990, one fourth of cars could be diesel-powered. Yet, a looming ban of diesel car sales could have severe economic implications for General Motors. In addition, diesels promised a fuel economy advantage of 25 to 30 percent over a comparable gasoline-powered car. Government officials thus considered diesel cars a crucial part of its energy policy during the Second Oil Crisis at the end of the 1970s.^8^

Notwithstanding its important locus in American energy policy, the increase in diesel car consumption also kindled concerns about their emissions’ alleged “adverse human health effects.”^9^ The EPA, the Department of Energy, and the Department of Transportation jointly approached the National Research Council (NRC) in 1979 in order to obtain scientific proof of the risks and impacts of diesel cars. Between 1981 and 1982, the Council’s Diesel Impacts Study Committee presented its report, which contained a preliminary assessment of diesel cars, its technological and engineering solutions to lower emissions, as well as epidemiological data and risk analysis on the health effects of diesel emissions.^10^

## Engineering Principles, Control Technology, and Political Measures

The NRC committee reviewed the available control technology that facilitated compliance with the proposed federal emissions standard for particulate matter—0.6 g/mi for 1982 and 0.2 g/mi for 1985. In addition, it highlighted the potential of future technologies. Further, research revealed that the quality of diesel fuel, the control system, and the limit of NO_x_ emissions directly affected the particulate emissions of diesel cars. In particular, a trade-off existed between NO_x_ and particulate emissions since exhaust gas recirculation, the primary control technology for NO_x_ emissions, increased the particulate output. Because particulate emissions were considered the main culprit in endangering public health, the US government agreed to waive diesel cars from the statutory 1981 NO_x_ standard of 1.0 g/mi and granted them an increased limit of 1.5 g/mi in 1981 and 1982. This political move enabled diesel car-producing automakers—in particular General Motors, the biggest manufacturer of diesel automobiles in the USA—to pass the 1982 0.6 g/mi particulate limit with minimal additional cost. Indeed, the range of zero to $ 30 per car reduced the economic impact of environmental legislation dramatically. Scientists and economists had also forecasted additional costs between $ 150 and 600 per car if the EPA pushed the particulate limit to 0.2 g/mi. The higher costs reflected different technological approaches. The latter standard was only attainable with an extra anti-pollution device, whereas a standard of 0.6 g/mi was feasible with only slight engine modifications.^11^

This hinted at the two different design options that, according to engineers, promoted lowering particulate emissions. One approach propagated an improvement of the combustion process within the engine. The other favored an after-treatment of exhaust gases: either a so-called reactor or thermal in-stream oxidation, a catalyst or a trap-oxidizer. This type of device was particularly relevant for vehicles weighing more than a ton in order to pass the scheduled standard of 0.2 g/mi for the mid-1980s. Yet, engineers conceded that no device had passed the durability test of 50,000 miles in 1981 and 1982. Moreover, the diesel committee predicted a lead-time of five years before mass production was feasible. This had a considerable impact on the political decision to enact the 0.2 g/mi standard and ultimately led to a postponement of its enactment until 1987, a time when hardly any diesel cars were sold in the USA.^12^

Already in the early 1980s, only one of the discussed technological approaches appeared “most promising.”^13^ According to engineers working at universities and in the industry, most notably for General Motors and Mercedes-Benz, this was the “catalyzed or uncatalyzed trap system,” the device that later became known as particulate filter.^14^ Engineers who developed the filter were guided by the principles they had applied to catalytic converters in the early 1970s. Particulate filters were thus considered an “outgrowth” of the technology used for converters. The appearance of Corning’s honeycomb structured filter, for instance, strongly resembled a catalytic converter. German suppliers like Eberspächer, a company which produced filters for Mercedes-Benz, used a similar design. Engineering journals such as *Automotive Engineering *and *Motortechnische Zeitschrift*, however, pointed out that the inbuilt characteristics and functional principles of the filter technology differed significantly from catalytic converters. The device, with its porous cell, was designed to filter the exhaust gases, and the particulates adhered to the honeycombed structured walls within the device. As progressively more particulates accumulated, the back-pressure increased, reducing both fuel economy and engine performance. A regeneration process therefore had to be initiated, periodically burning—or oxidizing—the particulates at temperatures between 480 and 600 Celsius every 50 to 100 miles.^15^

**Fig. 2:**
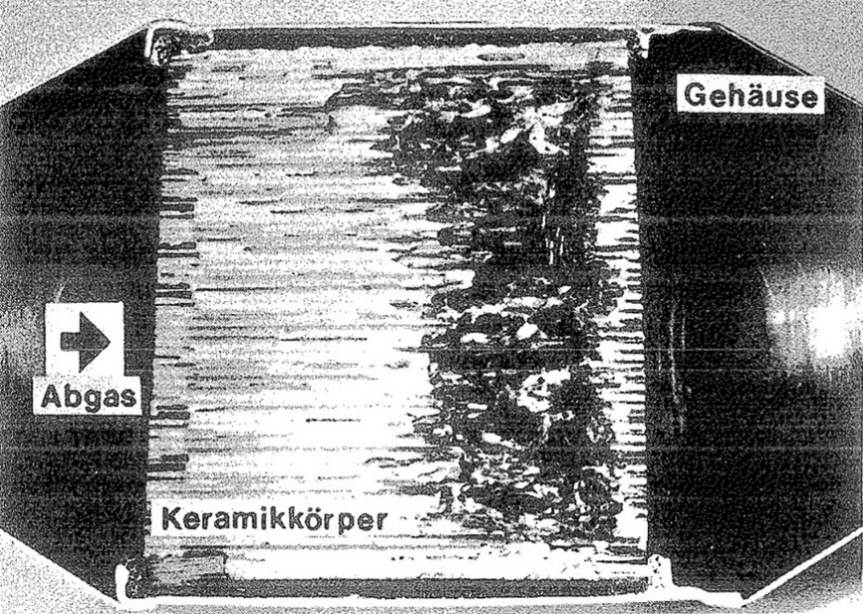
Melted Ceramic Monolith Particulate Filter After “Runaway” Oxidation. Exhaust flow enters the particulate filter on the left and passes the ceramic body that melted because of the high temperatures. (Source: Berg 1982: 298)

According to engineers working for automobile companies and suppliers, the initiation as well as the stabilization of the regeneration process—the burning-off of accumulated soot particles—was the most complicated design issue. Since diesel cars’ exhaust gas failed to reach the required temperature under regular driving conditions, the temperature of the exhaust gas flow had to be increased artificially. Engineers discussed several possible solutions of this problem. Eventually, they decided to use a combination of raising the engine speed, throttling, retardation, and exhaust gas recirculation.^16^ As the temperature increase could progress rapidly, the regeneration phase put the ceramic monolith under extreme stress. Guaranteeing its integrity over the lifetime of the vehicle thus proved to be the most demanding task—particularly as the surge could distribute unevenly, causing additional internal stress. In test procedures, the problem became evident when over-throttling induced complete failure of the device: first, the oxygen level fell below two percent, which increased the temperature but decreased the amount of oxygen needed for the combustion. When returning to the unthrottled driving state with reduced engine speed, enough oxygen was suddenly injected into the “superheated particulates on the trap.”^17^ The excess of oxygen precipitated a “‘runaway’ regeneration condition”^18^ and the particulate trap melted and destroyed the filter. This happened in particular right before the exhaust flow exited the filter as the temperature drastically increased within the trap, as shown in Fig. 2.^19^

However, Mercedes-Benz engineers showed that this contingency was very likely to happen under regular driving conditions. For instance, while driving at low speed in urban areas, soot accumulated on the porous walls within the filter. However, as the diesel car left city limits and accelerated on the freeway, the temperature of the exhaust flow increased rapidly, in turn generating abrupt oxidation that melted the particulate filter.^20^ Though the filters delivered the desired function of almost zero particulate emissions, they still failed in other areas: reliability, drivability, and durability. The National Research Council thus noted in 1982 that the use of particulate filters was “dependent on the development of reliable, durable, and marketable regenerative trap-oxidizers.”^21^ The engineers’ main task was therefore to find appropriate technological properties so that filters could be used in passenger vehicles.

## Technological Failure and Economic Impact

In 1982, GM engineer David Dimick summarized the results of his company’s research on particulate filters: Neither one of the 34 tested filter designs, nor any of the more than 100 materials used to construct them lasted the required lifespan of 50,000 miles. Industry critics claimed that GM had used a similar argument when they introduced the catalytic converter in the 1970s. But while a catalytic converter had suddenly become market-ready soon thereafter, automakers failed to introduce an operational particulate filter.^22^ It can be assumed that the quest for the best filter technology continued to be an lingering problem in the early 1980s. Engineers were still experimenting but unable to guarantee durability.^23^ All tested devices reduced particulate emissions, but only for a short period before failing. As General Motors and Volkswagen were unable to roll out “clean” diesel automobiles, one important factor beyond the diminishing reputation of low quality of US-produced diesels, consumer demand for diesel cars continued to fall in the USA. In 1981, General Motors and Volkswagen respectively produced about 350,000 and approx. 110,000 diesel cars, but by 1986 had reduced their output significantly to 588 and circa 18,000 cars.^24^

Only Mercedes-Benz was able to slow this trend because their diesel cars retained some of their positive image. Around 1981 and 1982, almost 80 percent of all Mercedes-Benz sold in the United States had been equipped with a diesel engine. Their share dropped to roughly 50 and 40 percent in 1984 and 1985. Diesels nevertheless remained an important cash cow and Mercedes-Benz thus hoped to secure market shares by introducing an allegedly market-ready filter in its most expensive and most powerful turbodiesel S‑class sedan in the fall of 1984. The car magazine *Road & Track* praised this move because it would rid the diesel of both, its cloud of black soot and its cancer-causing stigma.^25^

Two changes had occurred that forced the German automaker to introduce filter technology. First, the State of California—the most important market for Mercedes-Benz in the USA—set a different particulate standard by tightening the limit to 0.4 g/mi in 1985 and to 0.2 g/mi in 1986. Mercedes-Benz was only able to pass both thresholds with a particulate filter. Second, the fierce debate over the cancer-causing effects of diesel particulate emissions not only eroded the diesels’ reputation but also sparked a severe drop in sales (Neumaier 2020: 88–92). Mercedes-Benz envisioned that a diesel car with virtually no particulate emissions would be liberated of its daunting cancer stigma. Mercedes-Benz also hoped to regain their previous momentum and that diesel sales would recover. The company regarded fuel-efficient diesel technology an integral part of meeting the requirements of the Corporate Average Fuel Economy (CAFE) as well as a way to avoid financial penalties. As buyers of Mercedes-Benz cars shifted from diesel cars with high gas mileage to gas-guzzlers with Otto engines, the share of diesel cars in Mercedes’ portfolio further eroded to approx. 39 percent in 1985 and five percent in 1986. In absolute figures, in 1985 these were circa 35,000 total units, of which 31,000 were turbodiesels, and less than 3,000 units with 1,800 turbodiesels in 1986.^26^ This significant drop in diesel car sales corresponded in rising fines for failing to meet the CAFE requirements, reaching $5.5 million and $20.2 million in 1985 and 1986.^27^

Despite its limited availability, the particulate filter was portrayed in the media nationwide and Mercedes-Benz advertised the particulate filter as the “latest breakthrough” between 1984 and 1986 because it “cleaned-up” diesel emissions.^28^ A year later, media reports revealed that, despite the praise in advertising, Mercedes-Benz turbodiesels with particulate filters produced between 1985 and 1987 failed to perform on several levels. Some filters simply dissolved into pieces. Others were clogged by particulates or overheated during regeneration and, in some cases, even ignited the underbody of the car. The particulate filter thus not only brought into question the diesel’s reputation as a most reliable car, but also threatened the safety of its passengers. After voluntarily recalling 9,000 vehicles from the first generation in 1986/87, Mercedes-Benz rolled out an improved filter design that also broke down—at the latest after 30,000 miles. The ongoing problems proved to be a major engineering failure and public relations disaster, which eventually forced Mercedes-Benz to withdraw its most powerful diesel cars with particulate filters from the market by the end of 1987 (Neumaier 2010b: 162; Neumaier 2014: 446).^29^

This proved fatal for the most prestigious diesel car in the USA, as the failure of the particulate filter had a triply negative effect on the diesel’s reputation. Consumers perceived diesels to be unreliable—especially since GM-produced diesel cars already had a poor reputation, which stemmed from their catastrophic engine blow-ups in the early 1980s. The drop in engine power widened the performance gap between diesels and gasoline-powered cars. Car owners thus claimed once more that diesels were sluggish. In addition, a concerned public linked all diesel cars to cancer as the only remedy to their health threatening soot emissions—the particulate filter—had failed (Borg 2014: 295; Neumaier 2010a: 129–131; Neumaier 2010c: 40–43).^30^

Diesel cars sold in the USA thus failed to provide several functions that consumers demanded from automobiles due to technological constraints. Being labelled sluggish and unreliable was a major blow to diesel cars. But the link between diesel emissions and cancer was incredibly detrimental and sealed the demise of the diesel car in the USA during the mid-1980s. This image of diesel cars lingered and was so pungent that when Mercedes-Benz offered new models in the USA during the 2000s, car magazines consistently recalled their cancer-causing emissions, showing that they were still a major concern.^31^

## Ambiguities and Inconclusive Scientific Research Results

Environmentalists, but also car enthusiasts and prospective car buyers, branded diesel emissions as cancer-causing and potentially lethal. In contrast, the scientific discourse remained ambiguous, in particular during the first half of the 1980s when the EPA and the Carter and Reagan administrations discussed political measures against diesels. The US federal government relied on the expertise of the Diesel Impacts Study Committee and its reports presented an apparent consensus among members on the carcinogenicity of diesel emissions.^32^ “Materials moderately active as mutagens in various assays and as carcinogens when painted on the skins of susceptible animals have indeed been partially purified from diesel exhausts,” Philip Handler concluded in a letter to the EPA administrator Douglas Costle in September 1980. Handler, also the chairperson of the NRC and president of the National Academy of Sciences, then emphasized that “no evidence of carcinogenesis has been noted in animals breathing diesel exhaust fumes or in epidemiological studies of relatively heavily exposed human populations.”^33^ The “least common denominator”^34^ presented in the summary thus refrained from a critical assessment because unsettled and controversial viewpoints on health threats had been excluded from the report’s summary. Moreover, these weak and apparently inconclusive results allowed EPA and other US government officials to tend towards classifying diesel exhaust as “safe enough” to public health.

The Diesel Committee’s Health Effects Panel scientific risk assessment provided the backing for this decision, highlighting rather the uncertainties of scientific research than possible health risks. It had analyzed any available data on the health risks of diesel emissions in four different areas: mutagenesis, carcinogenesis, pulmonary and systemic effects, and epidemiology. Each field revealed myriad problems. For instance, some studies were “defective in some manner,” while others were still in progress, and therefore had not been peer-reviewed. “The effects of human exposure to whole diesel exhaust have not been conclusively demonstrated,”^35^ the Health Effects Panel further pointed out. This fact remained valid throughout the 1980s, since *in vitro* or *in vivo* studies only used organic material from animals such as hamsters, mice, and rats. The results obtained in these experiments were then used to predict possible effects on humans.^36^ Though this approach was in accordance with contemporary scientific standards, it always contained some uncertainty, because it is not possible to correlate one-to-one the results obtained in animal tests to the effects on human beings (Jasanoff 1992: 202–203; Langston 2010: 38).

This becomes apparent by analyzing results from studies on mutagenesis and carcinogenesis. Organic extracts of diesel exhaust particulates contained “mutagenic and carcinogenic potencies,”^37^ experiments indicated. The potency determines the intensity of mutagenic or carcinogenic effects that are categorized as weak, strong or very strong. In the early 1980s, doubts remained among scientists on how to classify the carcinogenic potency of diesel emissions.

Studies revealed that diesel exhaust extract contained carcinogenic materials, such as polycyclic aromatic hydrocarbons (PAH). For instance, the known carcinogen Benzo[a]pyrene was adsorbed by the carbon core of particulate emissions. When painted on animal skin, some laboratory tests with extracts of diesel fumes showed tumor formation. Experiments hence indicated “possible carcinogenic activity”^38^ of diesel emissions, but scientists were unable to obtain clear information on their precise mutagenic effect on mammalian cells. Toxicologists concluded that the results were “somewhat mixed”^39^ since tests with cells of hamster and mice showed both positive and negative results. These discrepancies, however, could have resulted from different set-ups, the Health Effects Panel assumed. In particular, the low dosage used in negative tests with hamster cells would also have shown no effects with mouse cells. Based on this weak data, scientists indicated that diesel exhaust particulate extracts contained mutagens that could also act as carcinogens. Yet, no evidence existed whether these cell mutagens were able to reach germ cells, and thus alter the genome.^40^

**Fig. 3:**
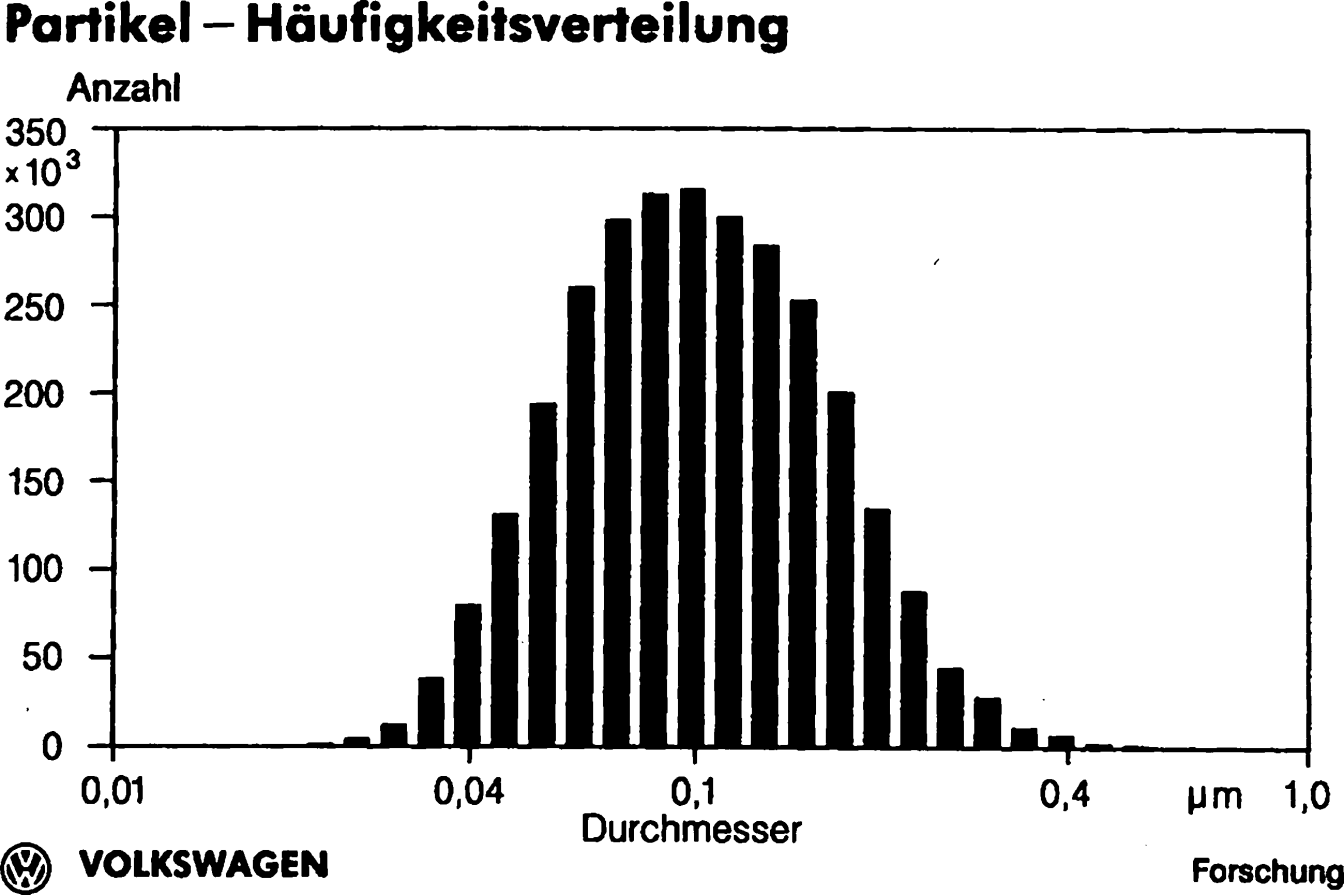
Particulates Size Distribution (diameter in µm and quantity). (Source: Klingenberg et al. 1991: 122)

Scientists and automobile engineers also knew that the particulates were of submicron size in the 1980s and 1990s. Ninety percent of the particulates were a size of less than 1 µm and the distribution maximum was at 0.1 µm (see Fig. 3). Since these tiny particulates could easily enter the respiratory tract, scientists also conducted inhalation experiments with animals. These studies, however, were still in progress at the beginning of the 1980s. Therefore, the Health Effects Panel drew only tentative conclusions: research revealed that “no convincing evidence [existed] that inhaled whole diesel exhaust is mutagenic or carcinogenic in laboratory animals.”^41^ Though diesel fumes could cause tumors when extracts were applied directly onto animal skin, inhalation experiments did not confirm these findings. Due to such contradictory results, scientists concluded that the carcinogenic activity of diesel exhaust was “low.”^42^

This viewpoint was shared among scientists, but their assessment on the potential health risks of diesel emissions diverged, in particular between the Diesel Impacts Study Committee and scientists working for the auto industry. While the former took a cautious approach, the latter tried to sow doubt in order to prevent political measures against diesels. The Health Effects Panel pointed out that animal testing provided vital information on how chemicals could affect humans. These tests were a crucial tool in assessing the health and cancer risks for humans. Once chemical compounds had been identified as carcinogens in animal tests, this should be seen as a “serious signal that a potential human health hazard exists,” the panel’s report highlighted.^43^

**Fig. 4:**
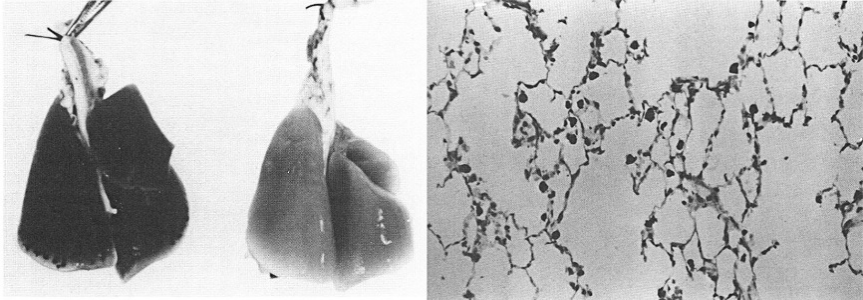
Exposed Lung (35 weeks to 1,500 µg/m^3^, *left*), Control Lung (*right*) and Microscopic Image of Rat Lung (2 weeks exposure to 6,000 µg/m^3^). (Source: Vostal 1980: 922–923)

Nevertheless, room for speculation remained since the precise effects of diesel emissions on human beings had not been disclosed. This void was filled with the interpretation of industry-sponsored scientific research—a type of counter-expertise to independent scientific research (Weingart 2005: 131). Jaroslav Vostal, the director of the Biomedical Science Department at General Motors Research Laboratories, concluded that “there was no immediate reason to regard wider use of diesel engines as a significant risk to human health.”^44^ He repeated his assessment before a public congressional hearing in October 1980. Extensive research in laboratories, according to Vostal, even provided scientific proof that cancer fears of diesel emissions were unfounded. This interpretation is striking insofar as Vostal’s own research showed how diesel fumes tremendously affected respiratory systems. After an exposure of 35 weeks with the highest concentration of 1,500 µg/m^3^, the lungs of rats turned black. Despite the visual display of the particulates’ impact on the lungs (see Fig. 4), Vostal claimed that only the appearance changed—but not the functionality. A microscopic analysis supposedly proved that a black-stained lung was still fully functional, as shown in another assay where rats inhaled a four-fold concentration of 6,000 µg/m^3^ for two weeks (see Fig. 4).

Vostal’s experiment setup, however, omitted one important methodological aspect of *in vivo* tests: tumor formation could only become detectable after extended exposure. Therefore, according to scientific standards, Vostal’s test set-up produced invalid results. Vostal even went one step further, claiming that a long and extensive exposure to diesel fumes “induces an active physiological defense process,”^45^ meaning the lung would clean itself of diesel particulates. Consequently, the risk for tumor formation would not increase, even with massive exposure to diesel exhaust fumes. Automakers used such allegedly scientific findings to bolster their argument that diesel particulates did not threaten public health.^46^

The approach taken by General Motors shows that the industry first tried to sow doubt concerning the carcinogenicity of diesel fumes by highlighting uncertainties in the effects of diesel particulates on human beings. Second, automakers initiated scientific research aiming to provide contradictory results to independent scientists in order to devalue their expertise with counter-expertise (for similar approaches of the industry see Oppenheimer et al. 2019; Oreskes & Conway 2010). Consequently, depending on one’s viewpoint, that is, political and economic interests, but also the research methods and experiments applied, the interpretations of carcinogenicity of diesel particulates could differ significantly. This triggered a quest for a quantitative method of risk assessment that provided indisputable scientific results, which could ultimately guide political decisions.^47^

The need for a more conclusive test became even more pressing when not only mutagenesis and carcinogenesis, but also epidemiology, provided inconclusive results and fostered further ambiguity. Hence, uncertainties remained on how to assess diesel fumes in 1981. The National Research Council wrote

in spite of the negative evidence that has been accumulated from epidemiological studies, it is possible that diesel exhaust is carcinogenic or mutagenic in animal or humans exposed by inhalation, but at level too low to be detected in studies conducted to date.^48^

Scientists continued to determine the critical threshold when diesel exhaust turned from “safe enough” to hazardous. Only additional research might have helped clarify the carcinogenic potency of diesel emissions, and therefore shed light on their health risks. This information, however, was not available.

## Cancer Risk Calculation Based on Weak Data

Politicians and the EPA needed guidelines for direct actions as the pressure for regulation mounted amid surging diesel car sales in the early 1980s. Where a state of scientific ambiguity on cancer risks existed, quantification provided the political tool to undertake these measures. The rigor of quantitative data and “objective” numbers appeared credible and therefore immune to criticism when being contested in hearings before bodies such as a court or the House of Representatives. Risk analysis eventually became the most influential quantitative method in the 1980s—a time when not only the political climate but also the trend in science leaned towards a mathematization (Brickman et al. 1985: 304; Jasanoff 1990: 58; Porter 1995: 8, 196–198).

In case of the diesel, the Diesel Impacts Study Committee provided this relevant piece of data in 1981/82. The committee published its risk assessment prior to the National Research Council’s report, which reviewed *Risk Assessment in the Federal Government* in 1983. It argued in favor of differentiating between risk assessment and risk management. The former deals with scientific research results; the latter with the implementation of regulatory decisions into politics, the legal system, and economics. Due to the unproven link between diesel particulates and cancer, the committee recommended postponing the tightening of the particulate standard to 0.2 g/mi until after the mid-1980s. The US government supported this approach. This decision is significant: the lack of definitive scientific proof and estimates on cancer risks allowed officials to place economic considerations over potential health risks. Yet, the National Research Council also advised the EPA to revise this decision once more scientific knowledge was available.^49^

In this state of uncertainty on the carcinogenicity of diesel particulates, researchers nevertheless tried to assess the impact of diesels on public health because they wanted to provide guidelines for political regulation. Cancer risk describes the probability of cancer formation after being exposed to a certain dosage of a chemical compound. This method of risk assessment uses exact numbers, for example 1 in 100,000 exposed persons will develop cancer.^50^ Calculating such exact numbers became a complicated task because epidemiology lacked solid data in the early 1980s. Several studies nevertheless tried to assess the cancer risks of diesel emissions. For instance, the economist and physician Jeffrey E. Harris published a report for the Diesel Impacts Study Committee in 1981. Two years later, an abridged version of Harris’ report appeared in a special issue of the journal *Risk Analysis*, published by the Society of Risk Analysis. Founded in 1980, this society became the platform of scientists discussing probabilistic calculations that allowed estimating potential cancer risks, eventually institutionalizing risk analysis as a scientific discipline. Risk analysis additionally became a tool for justifying political measures. Robert B. Cumming, a toxicologist at Oak Ridge National Laboratory in Tennessee and one of the founders of the society, noted in the special issue of *Risk Analysis*:

Most risk assessments are limited to the estimation of risk factors that would apply under specified conditions, and make no attempt to project what is likely to occur at some time in the future. But regulatory decisions that are made today are an attempt to deal directly with tomorrow’s hazards.^51^

The case study of the health risks of light-duty diesel vehicles was, according to Cumming, one prime example. It allowed analyzing how risk assessment in the present could affect political decisions and thereby future technological developments. One crucial obstacle remained: how to assess and calculate cancer risks was still an unresolved issue among researchers. As industry-sponsored research fostered doubt and provided contradictory results on the carcinogenicity (for this general approach see Oreskes & Conway 2010), political regulation of diesel exhaust came under pressure. In particular due to several uncertainties (such as a limited data base, obscurities about the model selection, as well as future engineering approaches and political measures) independent research tried to develop sound methods of calculating potential health risks. Only that would allow to provide a solid estimate of health and cancer risks. Based upon this, politicians could then decide whether the risks—as well as the estimated number of sick and dead people—would be “acceptable,” or if political action was mandatory. Nevertheless, the health risks of diesel cars had an impact on politics, before it was possible to provide sound scientific evidence on which the cancer risks could be calculated. The special issue of *Risk Analysis* reflected on this issue and tried to evaluate different problems in hindsight to provide better estimates for future political decisions. Cumming considered this mandatory as he concluded in his introduction to the issue: “We try to project future trends and attempt to estimate their risks because we must.”^52^

Harris conceded that none of the completed epidemiological studies satisfied all scientific criteria. They either lacked a well-defined social group or the evaluation of different factors or a solid duration of exposure.^53^ Harris nevertheless used the available epidemiological studies and estimated a “potential risk equal to a 0.05% proportional increase in lung cancer incidence per unit of cumulative lifetime exposure.”^54^ One unit of exposure, according to Harris, matched inhaling a concentration of one microgram or particulates per cubic meter (1 µg/m^3^) for one year. Based on this estimate, Harris calculated the risk of an increase in lung cancer incidence to 1 percent, if a male aged 40 to 65 years inhaled an average of 1 µg/m^3^ for 20 years. The risk jumped to 15 percent if the same test person inhaled an average of 10 µg/m^3^ for 30 years. Harris compared the cancer risks of diesel emissions to the potential risks of other chemical compounds, in particular cigarette smoke and asbestos. Both showed much higher cancer risks. The rate increased by 100 to 700 percent for a male non-smoker if exposed to asbestos for 20 to 30 years. A male smoker aged 40 to 65 had an increased cancer risk in the range of 1,000 to 2,000 percent if he smoked cigarettes for a comparable period.^55^

Harris’ calculation revealed a comparatively low cancer risk from diesel particulates compared to other chemical compounds. Several scientists claimed that his estimates and calculations were rife with uncertainties. For instance, researchers from the Inhalation Toxicology Research Institute, later called Lovelace Respiratory Research Institute and most recently renamed to Lovelace Biomedical, raised concerns. Scientists from this private, non-profit biomedical research institute compared three different cancer risk estimates in 1983, which previously had been used by US government. Richard Harris had developed two of these models, which differed significantly in the estimated cancer risks. EPA scientists used a third model based on a comparative potency method for cancer risk assessment.

The variant results of potential cancer risk become apparent when linking the models to concrete figures: based on a population of 230 million US citizens exposed to 1 µg of diesel particulates per cubic meter over a lifespan of 70 years and an average of 100,000 lung cancer deaths per year, the EPA scientists’ model yielded that diesel particulates would cause 100 lung cancer deaths. Harris’ second model estimated the cancer risk at 0.0035 percent and thereby yielded a similar figure of 245 deaths. However, his first model, presented above, calculated 3,500 deaths per year and presented a significant deviation. The scientists concluded that “epidemiologic studies […] may not provide sufficient insight into risk assessments for populations such as that of the United States to define risks from atmospheric pollutants for the regulatory process.”^56^ Despite obvious shortcomings, US government officials had previously still relied on epidemiological studies to estimate cancer risks, such as Jeffrey Harris’ report to the Diesel Impacts Study Committee, which demonstrated a very low incidence. Harris’ findings formed then, among others, the basis for the Reagan Administration’s political decision and EPA administrator Anne Gorsuch’s industry-friendly policy to not regulate diesel emissions.^57^

The committee used Harris’ estimates and formulas to calculate the “relative risk of lung cancer” of diesel particulates. The applied equation read “1 + r × C × D.” Specifically, “C” signified the ambient concentration of particulates in µg/m^3^; “D” designated the duration of exposure in years; and “r” represented the estimated parameter, based on epidemiologic and laboratory evidence. Harris estimated the latter to be 0.0005 (or 0.05 percent). The formula thus calculated an increase in “relative risk of lung cancer” for a middle-aged man exposed to 2 µg/m^3^—twice the concentration of exposure used by Harris, as mentioned above—over 20 years to two percent. Smokers and workers exposed to asbestos were once more the point of reference for the cancer risk assessment: a smoker suffered from a ten- to twenty-fold increase in lung cancer risk and a man exposed to asbestos a two to eight-fold increase.

The potential lung cancer risk of diesel particulates appeared less daunting. To put these relative numbers in absolute figures: the annual death rate of lung cancer for male Americans aged 55 to 64 amounted to approx. 220 per 100,000 individuals. Between 180 and 200 of these deaths were directly linked to cigarette smoke. Only four persons in 100,000 (two percent of 220 = 4.4), according to Harris’ calculation, suffered from lung cancer due to diesel particulate emissions.^58^

According to these calculations, the Diesel Impacts Study Committee and US government were able to establish the cancer risk of diesel particulate emissions as negligible in the early 1980s. When Anne Gorsuch (1942–2004) succeeded Douglas Costle (1939–2019) as EPA administrator, she could base her lenient policy towards diesel cars on these scientific estimates. Her approach was also part of the general policy of the Reagan Administration favoring deregulation in order to downsize government and to initiate technological innovation and thereby economic growth. In addition, the general risk of diesel exhaust fumes decreased after 1981—diesel car sales peaked in the USA that year. Dwindling sales resulted in declining particulate emissions, thus reducing their threat to public health. In contrast to the federal government, the public still continued to consider diesel particulates a cancer threat. Environmentalists even argued that diesel emissions were as lethal as cigarette smoke and therefore had to be banned. This assertion, however, contradicted official policy that based its cancer assessment, among others, on Harris’ calculation.^59^

The State of California also opposed a lenient policy towards diesel emissions, which was in line with the general approach towards health threats of the California Air Resources Board (CARB). In September 1982, the chairperson of the CARB, Mary Nichols (1979–1983, 2007–probably 2020), announced that she aimed to reduce drastically the threat of airborne cancer-causing chemicals because of the poor air quality in Los Angeles County. The strategy of minimizing the “unnecessary risk of cancer” targeted benzene, carbon tetrachloride, chloroform, vinyl chloride, and particulate matter, among others. California’s approach thus did not specifically target diesel cars but, nevertheless, diesel emissions were listed as a primary source of carcinogens. Furthermore, Nichols opposed a threshold approach to identify levels of toxicity, an argument used by industry representatives to defend against regulation of hazardous materials in the 1970s and 1980s “because scientists could not agree whether there was any level low enough to be completely safe.”^60^ In contrast to the federal approach, where consensus on a “least common denominator”^61^ had been established and prevented harsh regulation of diesel emissions, the CARB applied the precautionary principle. This approach permitted imposing the stringent particulate standards that had pressured Mercedes-Benz to introduce the ill-fated particulate filter. The CARB’s position can be explained for one thing with the higher air pollution in the Los Angeles Basin compared to the rest of the USA, and for another with the fact that no economic interests were at stake, as carmakers did not have any major plants in California. The economic pressure applied by California’s market has nevertheless been massive: from 1977 to 1985, residents of California purchased between eight and ten percent of all cars and light truck sold in the USA.^62^ The critical attitude towards diesel cars continued into the 1990s and persisted long after diesel cars had virtually disappeared from California. In 1992, the California State Environmental Agency made this once more unequivocally clear: “We don’t want Diesel. We want clean air.”^63^

While this negative perception of diesels prevailed in the 1990s, the disdain for diesel cars began to slowly shift in the early 2000s—at least among the more eco-conscious California consumers. Diesel cars’ appreciation increased because their lower fuel consumption promised to reduce carbon dioxide emissions and thereby global warming. Furthermore, engineers considerably improved the diesel emissions by introducing both a functional particulate filter and a selective catalytic reduction of nitrogen oxides emission. Consequently, diesel cars met not only California standards for nitrogen oxide emissions but also their cancer-causing particulates had been virtually eliminated by operational particulate filters. Yet, as Mary Nichols pointed out in 2017, the VW diesel scandal revealed by both CARB and the EPA shattered the slowly rehabilitated image of diesels.^64^

## Classification of Diesel Fumes as Carcinogenic in Germany 1986–1989

The political pressure to evaluate the cancer risks of diesel emissions vanished once sales of diesel cars started to tumble in the USA in 1981 for two reasons. The first US mass-produced diesel cars by General Motors suffered from major engine problems, but also Volkswagen and Mercedes-Benz diesels gained the reputation for being unreliable because of breakdowns. In addition, the dangers of cancer that had been linked to diesels scared prospective car buyers away from them, and diesel owners were desperate to sell their vehicles (Neumaier 2010b: 129–131; Neumaier 2014: 442–446).

By contrast, in Germany and Europe diesel emissions did not become a major concern until the mid-1980s. In West Germany, the share of diesel cars among new car registrations jumped from 4.8 percent in 1977 to 15.1 percent in 1982, later peaking at 27.4 percent in 1986 (see Fig. 1). The general acceptance of diesel cars increased in Germany due to a combination of several factors. This not only included their low fuel consumption but also their improved drivability. Moreover, they had been labelled the eco-friendly alternative to the gasoline-powered car, which still ran on leaded gasoline and without catalytic converters. This rise in diesel car sales correspondingly impacted the number of diesel cars travelling the roads in Germany, which in turn multiplied diesel emissions. In 1985, 5.3 million registered diesel cars emitted 45,000 tons of particles in Germany. Forecasts predicted that diesel cars could account for up to 15 million vehicles by the mid-1990s. The annual particulate emissions could subsequently reach 107,000 tons.^65^

Moreover, the differences between diesel and gasoline-powered cars became evident in the second half of the 1980s. Gasoline-powered cars with catalytic converters and unleaded gasoline were introduced in 1985. Initially, only a few gas stations offered unleaded gasoline. And few car owners refueled their old vehicles with unleaded gasoline because rumors circulated that it severely affected drivability and durability. Until mid-1986, most car buyers shied away from purchasing gasoline-powered cars with catalytic converters. They preferred diesels that had a reputation for being sturdy, reliable, and fuel-efficient. In addition, the German government had labelled diesel cars “low-emission,” and thus granted them tax subsidies.^66^

Yet, the unanimous praise for diesel cars began to fade in the autumn of 1986. Car owners and buyers started to change their minds, once the new types of fuel and cars gained broad acceptance. A growing number of drivers refueled their vehicles with unleaded gasoline and consumers shifted towards cars with catalytic converters. As previously in the USA, particulate emissions suddenly became a peculiarity of diesel cars. Moreover, these particulates simultaneously turned into a debated pollutant since they had gained a notorious reputation for being hazardous. In addition, research on their carcinogenic potential intensified, and the political pressure for regulation increased in Germany too (Neumaier 2010b: 169, Neumaier 2014: 446–449).

Once again divergent views clashed over the question how many of the 26,000 lung cancer deaths per year (out of circa 160,000 cancer deaths) could be attributed to diesel cars.^67^ Even some of the most ardent supporters of the automotive industry, the car enthusiasts’ magazines such as *Auto Motor und Sport*, switched sides. In 1987, the magazine declared diesel exhaust fumes a “rat poison”^68^ and harshly criticized lax European emission standards. The ADAC (*Allgemeiner Deutscher Automobil-Club*), Germany’s largest automobile association, had already started to question that diesel emissions were “safe” for the public health in 1984. Only after German car owners accepted unleaded gasoline and gasoline-powered cars with catalytic converters did public opinion capsize, as notably indicated in the critical remarks by car magazines.^69^

Several institutions and researchers contributed the scientific backing for this belief. At a hearing of the West German Federal Environment Agency (*Umweltbundesamt*; UBA) in November 1986, scientists from the Fraunhofer-Institute for Toxicology and Aeorosol Research in Hannover, the Battelle Institute in Geneva, and the Medizinische Institut für Umwelthygiene in Düsseldorf argued unanimously that diesel fumes were carcinogenic, and thus hazardous to human health. In 1987, the *Senatskommission zur Prüfung gesundheitsschädlicher Arbeitsstoffe *(*MAK-Kommission*) of the German Research Foundation (DFG)—the committee that defined the maximum allowable concentration of pollutants at the workplace—moreover labelled diesel emissions a health threat, and set a maximum allowable concentration in the workplace. This critical view of diesels threatened their future, and in turn the profits of the diesel-producing automotive industry—in particular Volkswagen and Mercedes-Benz—especially since consumers (increasingly) shied away from purchasing diesel-powered cars. Their share in new car registrations tumbled from 27.4 percent in 1986 to 10.4 percent in 1989 (see Fig. 1).^70^

The consensus within the German scientific community enabled political decision makers to take a critical stance towards diesel. The UBA, however, adopted this viewpoint only initially, and considered diesel emissions carcinogenic for merely a short period. In 1986/87 the Environment Secretary, Walter Wallmann (1932–2013), intervened and pressured the UBA administrator Heinrich von Lersner to withdraw a critical assessment of diesel emissions. Consequently, the UBA issued a press release stating that diesel emissions—in particular those of automobiles—posed no threat to public health. Wallmann’s successor Klaus Töpfer continued his pro-diesel position in office. In short, the UBA continued to consider diesel emissions as risk-free for the public health.^71^

This assessment did not affect diesel car sales immediately, but it did create an opportunity for German automakers to shed doubt on the carcinogenicity of diesel emissions. The German Association of the Automotive Industry (*Verband der Automobilindustrie*; VDA) published a public relations brochure aimed at taking the wind out of diesel bashers’ sails. The industry compared emissions of gasoline- and diesel-powered cars. According to the auto industry, the latter appeared to be superior because—in total—diesels emitted fewer pollutants and consumed less fuel and thereby gave off fewer carbon dioxide emissions. In particular, the diesel-powered vehicles became relevant when the greenhouse effect and global warming became political concerns in Germany at the end of the 1980s (Neumaier 2010b: 197–200; Neumaier 2014: 452; Vogel 2012: 129).^72^

The German automotive industry not only tried to establish a counter-expertise—like their US counterparts as indicated above. They also actively discredited established experts in its brochure, suggesting that renowned scientists, such as Hans-Werner Schlipköter (1924–2010) of the *Medizinisches Institut für Umwelthygiene*, had conducted “bad science.”^73^ Supposedly, he had made methodological errors in his experiments—an allegation scientists vehemently opposed. The VDA claimed that animal tests with hamsters, mice, and guinea pigs had not provided any evidence on the carcinogenicity of diesel fumes. Only tests with rats had shown tumor formation, but only when unrealistic concentrations of diesel fumes had been used. The level was 300-fold higher than on even heavily travelled roads. Furthermore, rats had to be exposed to these concentrations five days a week for 16 hours over their lifespan in order to show cancer signs. At a concentration of 100 to 200-fold of the average level, rats did not show any response, the VDA emphasized. A statistically significant higher cancer risk was only reported at a concentration of more than 1,000-fold the average limit of road areas.^74^

The VDA concluded that diesel fumes had to be considered “safe” to human health since rats did not show any signs of tumor under normal road conditions. Furthermore, the classification as carcinogenic was unfounded because, according to epidemiologists, 90 percent of the lung cancer risks were caused by cigarette smoke. The second most common source of lung cancer was pollution in the workplace. Air pollution, according to the VDA, only ranked third. The VDA aimed to dismantle scientific consensus on the risk assessment of diesel emissions by discrediting scientists and insinuating that unsound methodological approaches had been taken, leading to flawed research results. By creating casting shadows of uncertainty about the cancer risks of diesel emissions, automakers and their lobbying institution wanted to dispel growing cancer fears of diesel emissions that had reached a peak in the West German public debate.^75^

Scientific research did not back up the auto lobby’s arguments. Unlike the early 1980s, when only inconclusive results had been available, research strongly indicated that diesel exhaust particulates had to be considered a health threat by 1987/88. Besides the MAK-Commission, the International Agency for Research on Cancer (IARC) at the World Health Organization (WHO), and the U.S. National Institute for Occupational Safety and Health (NIOSH) declared diesel exhaust a cancer-causing agent.^76^ Eventually, even the German government adopted this viewpoint—albeit only for a short-lived period between 1988/89, thereby infuriating German automakers even though a political ban of diesel cars had never been discussed (Neumaier 2014: 449; Neumaier 2020: 97).

At a research colloquium of the *Verein Deutscher Ingenieure* (VDI) in 1991, the association of German engineers, the diverging viewpoints clashed. Friedrich Pott, employed by the *Medizinisches Institut für Umwelthygiene* in Düsseldorf, rejected the auto industry’s arguments. He and his colleague Uwe Heinrich at the Fraunhofer Institute in Hannover had published several scientific papers pointing out the carcinogenic potential of diesel fumes based on inhalation experiments with rats. In particular, rats exposed to a concentration of 2.2 to 7.1 mg/m^3^ particulates over a period of 35 to 96 hours per week for 2 to 2 ½ years, showed a rate of lung tumor development ranging between 3.6 and 38.5 percent, while the control group of non-exposed rats reached a rate between 0 and 1.4 percent. These results indicated that diesel particulates led to a statistically significant higher cancer risk. In addition, Pott and Heinrich argued that animal testing afforded a considerably more accurate basis for the estimation of cancer risks than the epidemiological studies used by EPA cancer risk assessment. This suggests emerging different scientific approaches in the assessment of cancer risks in Germany and the USA. While during the 1980s cancer risk in the USA relied more on epidemiological data and risk analysis than on animal tests, German toxicologists continued to use both, epidemiology and animal tests. Notably, scientific evidence on the carcinogenicity of diesel particulates initially relied mostly on the latter approach in the second half of the 1980s. Around 1990, epidemiological research also strongly indicated that diesel emissions were carcinogenic.^77^

In due course, Pott and Heinrich became targets of the automakers’ criticism that was once again directed at doubting the carcinogenicity of diesel particulate emissions. Engineers usually deployed two arguments to denounce medical and biological research on the cancer-causing effects of diesel fumes, such as the previously published VDA brochure. First, they argued that one cannot infer test results from animals on human beings. Second, they further contended that the concentration of diesel fumes was too high and therefore unrealistic. Pott countered these hypotheses. He argued that in 80 percent of the cases animal tests provided the scientific basis for the classification of compounds as carcinogenic; results from epidemiological surveys were used in 20 percent of the cases. This approach was in line with scientific standards because the carcinogenic effects of substances in animals, such as rats, correlated with tumor formation in humans. Pott also conceded that, nevertheless, definitive scientific proof of the carcinogenicity could not be furnished.^78^

Human experiments were disqualified for ethical reasons, Pott implicitly emphasized. Second, the dosage of diesel exhaust fumes, Pott and Heinrich pointed out, was only 100-fold higher, if the reference point was a human being exposed to low levels of diesel fumes. The auto industry had taken this position in their public announcements. Environmental health and occupational medicine professionals opposed such an approach. Instead, Pott and other scientists considered the highly-exposed person as the point of reference. If the average exposure to diesel particulates in housing areas next to busy roads fell within the range of 15 µg/m^3^ over a period of 168 hours per week, then Pott used a dosage with a factor of 50–65—and not the alleged 300 to 1,000-fold higher concentration as claimed by the auto industry. And if the exposure to diesel particulates in a workplace reached 500 µg/m^3^ over 40 hours per week, Pott applied a 6 to 10-fold dosage in his experiments. Biologists and physicians considered this a common set-up for laboratory experiments; in order to trigger cancer with a low number of 50–100 laboratory animals, an over-dosage of diesel particulates had to be employed. Finally, Pott and Heinrich informed detractors that inhalation experiments at five different institutions in four countries had posted similar results: rats developed tumors after inhaling diesel particulates. In Germany, all expert committees and federal agencies had classified diesel fumes as carcinogenic.^79^ Pott also shed light upon the potential cancer risk of diesel particulates: he estimated the risk for the formation of lung tumors with lifelong exposure to 10–15 µg particles per m^3^, the average concentration in housing areas next to busy roads, to 70–100 incidents per 100,000 exposed persons. Pott argued that, in comparison to other cancer-causing atmospheric pollutions, this risk was “high.”^80^

## The Political Turnaround to “Clean” Diesel Emissions vs. Scientific Evidence on Carcinogenity

The scientific evidence appeared to be indisputable—yet the public controversy went on nevertheless. The VDA, Volkswagen, and Mercedes-Benz repeatedly opposed the scientists’ arguments and questioned the carcinogenicity of diesel exhaust, arguing that the results had been obtained by “unscientific”^81^ methods. The labelling of diesel fumes as a “killer substance”^82^ was, according to automakers, a “fearmongering” campaign of both the UBA and German government. The drop in diesel car sales, which in turn eroded the automakers’ profits, was the driving force behind this line of argumentation. In addition, engineers reacted and presented new technological devices that were supposed to “clean” diesel emissions and make them “safe” for public health.

Volkswagen and Mercedes-Benz addressed the issue similarly. They optimized combustion within the engine and installed an oxidation catalyst. Volkswagen, in addition, used a turbo-charger, while Mercedes-Benz picked exhaust gas recirculation as an additional measure. The head of Volkswagen engine development, Peter Hofbauer, clearly favored reducing particulate emissions by improving the combustion process within the engine. He returned to design principles that had been discussed in the 1970s, but were not considered appropriate in the early 1980s—the prime of the particulate filter. Hofbauer even opposed filter technology outright.^83^

Engineers at Mercedes-Benz shared his viewpoint, remembering the failure of the device on the US market. Engineers from Mercedes-Benz and Volkswagen also pointed out that diesel cars met European particulate standards, after modifying the combustion—an internal improvement of the diesel engine—and either installing a turbocharger or an exhaust gas recirculation. In 1989, Volkswagen presented its VW Golf “eco” diesel and Mercedes-Benz their “Diesel ’89”-engines. As these new “clean” diesel cars hit the market, the CEO of Daimler-Benz, Edzard Reuter, claimed the “real risk” for lung cancer was “nil.”^84^ Car magazines bought into this idea, once more flipped sides, and in turn mediated this viewpoint to the public. In addition, the German government—after private negotiations of then chancellor Helmut Kohl (1930–2017) and the Secretaries of Economic Affairs, Transportation, and Environment with representatives of the auto industry—revoked its critical assessment of diesels and introduced a particulate limit of 0.08 g/km (equaling 0.129 g/mi). The so-called “Töpfer-Norm,” named after the Minister for the Environment, became the new point of reference. It was a political threshold not based on scientific results: it could be publicly endorsed as “clean” and “safe” since it was lower than the US standard of 0.2 g/mi. In the German political debate, this limit was used as a benchmark, even though the California limit of 0.08 g/mi was far more severe (introduced in 1989). Hence, the “Töpfer-Norm” hardly represented a precautionary principle. It was not a “technological” threshold because most of the diesel automobiles produced by Volkswagen and Mercedes-Benz surpassed this “demanding” limit. German government officials supported automakers and, in addition, favored diesel cars by granting the sticker “particularly low-emission,” which was connected to tax incentives. This political move aimed at reducing the economic pressure on diesel manufacturers, which had suffered the economic impact of a severe decline in sales (see Fig. 1). In the early 1990s, the political initiative, among others, was one factor that contributed to a growing demand in diesel car sales.^85^

**Fig. 5:**
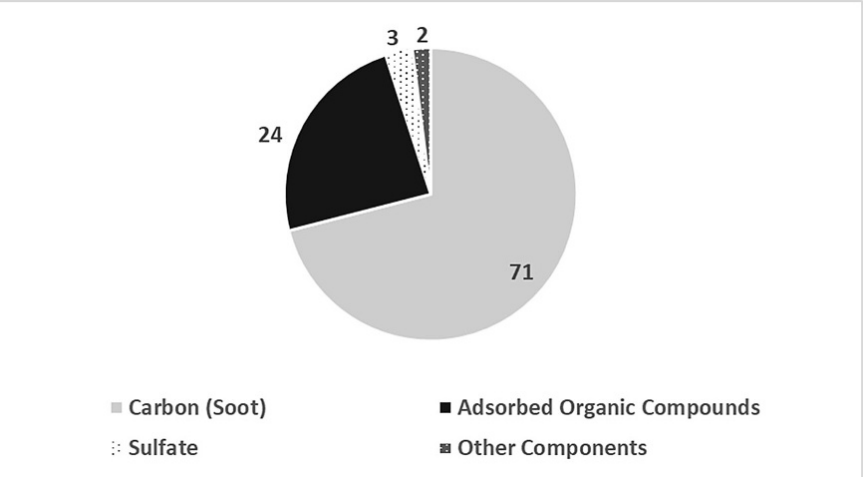
Composition of Diesel Particulates. Carbon (soot) and adsorbed organic compounds such as PAH and HC yield 95 percent of the total mass of particulates. (Source: Klingenberg et al. 1991: 127)

The approach to clean diesel emissions by installing an oxidation catalyst appeared to be logical from an engineer’s viewpoint but not from the scientists’. The device oxidized the HC and PAH emissions adsorbed to particulate emissions and thereby reduced the mass of emitted particulates, but, significantly, not the amount of emitted particulates. Diesel particulates comprised up to 71 percent of pure carbon and up to 24 of adsorbed organic compounds such as hydrocarbons (see Fig. 5). Technically, the catalyst could lower the emitted mass of particulates by one fifth to one fourth and thus pass even the new federal standard in Germany, which weighed the amount emitted.^86^

The newest contemporary research in the fields of biology, medicine, and toxicology, however, shed a different light on the engineering solutions to clean diesel emissions. Research results had already indicated in 1987/88 that the particulate core—and not the adsorbed organic compound such as the PAH, most notably the known carcinogen benzo[a]pyren—triggered cancer. Pott and Friedrich both supported this viewpoint. They argued that the low dosage of the PAH in the diesel exhaust could not stimulate tumor formation. Therefore, the mass of emitted soot—or carbon—determined the carcinogenic potency of diesel emissions. Further research substantiated these initial findings until studies then demonstrated that the carbon core of diesel particulates became the cancer-causing agent. In turn, these results boosted the reputation of gasoline emissions, which also contained PAH, but hardly any particulates. Therefore, gasoline-powered cars had to be considered “safe enough,” once a car had been equipped with a catalytic converter and ran on unleaded gasoline.^87^

Pott’s and Heinrich’s results further indicated that the cancer-causing effect was not linked to specifically diesel particulates but to any form of soot or particle. Their carcinogenic potency correlated with their size and surface: particles of a smaller diameter and a bigger specific surface of the inhaled particle showed a higher cancer-causing effect.^88^ Based on their results, Heinrich and Pott attacked the auto industry, claiming that the control technology presented by Volkswagen and Mercedes-Benz was ineffective. The oxidation catalyst only oxidized the PAH, but neither affected the number of particles nor the mass of the carbon core. Therefore, this device did not reduce the carcinogenic risks of diesel emissions. Quite the contrary, as it made diesel particulates smaller and thus more respirable.^89^

However, in the early 1990s, this viewpoint was still contested among scientists, and automakers once again capitalized on the uncertainty by casting doubts and preventing a consensus on the risk assessment of the carcinogenicity of diesel emissions. In fact, sometimes divergent interpretations even emanated from one institution such as the Fraunhofer Institute for Toxicology and Aerosol Research. The former head of the institute, Werner Stöber, claimed that, according to the research conducted, among others, by Uwe Heinrich, diesel fumes could not be classified as a “probable carcinogenic” agent. The results indicated that the carbon core of particulate caused cancer and that this effect was specific to rats and not other animals such as dogs, cats, and guinea pigs.^90^

The implication was that it would be impossible to use these results to predict cancer risks for humans. Furthermore, they contended that particulate concentrations used in the laboratory test were too high and therefore did not resemble real life conditions. Stöber not only repeated arguments that had been debated throughout the 1980s, but also became the scientific authority that supported the auto industry’s viewpoint. The UBA, however, did not adopt his opinion. Rather, it relied on Pott’s and Heinrich’s expertise, in particular because, besides animal tests, epidemiological studies also stressed the carcinogenicity of diesel fumes. Other research institutions continued to rank diesel emissions as a carcinogen. For instance, the Health Effects Institute in Boston published its widely received critical analysis of the health risks of diesel emissions in 1995.^91^

Yet, one problem remained: scientists could still not provide definitive scientific proof of diesel particulates’ carcinogenicity. They remained a “probable” or “highly probable” carcinogen. This ambiguity kicked once again the door open for speculation. Exemplarily, this effect can be highlighted with results of the Health Effects Institute. The auto industry argued that the institute’s findings indicated only weak cancer potency as the risk of lung cancer only increased 1.2 to 1.5-fold after exposure. The UBA in turn referred to the introduction of the report: “Diesel emissions have the potential to cause adverse health effects.”^92^ This included cancer and cardiovascular diseases.^93^ As the actual impact on public health could still be interpreted differently, diverging viewpoints lingered. In the early 1990s, the political pendulum swung in favor of the auto industry—despite the UBA’s and the scientists’ warning of the hazardous diesel particulates. Global warming was one crucial factor that facilitated the shift back to labelling diesel emissions “clean”. It had become one of the most important topics in German environmentalism. Suddenly, the fuel-efficient diesel car appeared in a different light. Its lower carbon dioxide emission, in comparison to a gasoline-powered car, could be labelled “eco-friendly,” while the cancer-causing effects of diesel particulates vanished from media coverage.^94^

## Conclusions

The analysis of the triangular relationship between technology, science, and politics revealed how the coding of diesel emissions as “safe” or as “hazardous” emerged and shifted in the US and in West Germany between 1977 and 1995. The political thresholds of emission limits not only differed significantly on both sides of the Atlantic but the coding itself was embedded in specific temporal, intellectual, and geographical contexts.

First, diesel fumes came under review in the USA during the late 1970s—about ten years earlier than in Germany. In particular, one chemical compound of diesel exhaust attracted new attention—particulate matter. At that time, the Carter Administration considered fuel-efficient diesel cars an important part of its energy policy. Research on the carcinogenicity of diesel particulate emissions was in its nascent phase. Therefore, scientists could only present preliminary and inconclusive results on the health effects of diesel particulates. They thus issued a cautious warning that diesel particulates might be carcinogenic. Industry-sponsored research countered this assessment with diverging results that negated the link between diesel particulates and cancer. From this viewpoint, diesel fumes were harmless. General Motors endorsed this counter-expertise in scientific journals and congressional hearings. This strategy thus spread doubt, which was a common industry approach to avoid regulation (see Oreskes & Conway 2010). Simultaneously, independent research was unable to prove the carcinogenicity of diesel particulates. Rather the contrary, risk assessment only calculated a “low” carcinogenic effect of diesel particulates. The combination of inconclusive results, numerous doubts, and a low potential cancer-risk enabled US government officials in the process of risk management to classify diesel fumes as “safe enough” to public health. As Sheila Jasanoff has shown with other recently discovered new chemical compounds (Jasanoff 1990), US government favored economic costs over threats to public health.

When diesel particulate emissions became a major concern in Germany after the mid-1980s, automakers had to follow a different strategy. Then, scientific research had undeniably shown that particulate matter could act as a cancer-causing agent. Thus providing counter-expertise did not suffice—in particular as the German government and the general public as well as car magazines unanimously considered particulates a carcinogen. Representatives of the automotive industry and its lobbying groups hence tried to influence the opinion towards diesel in a different way: they argued that the toxicologists’ results were highly questionable because they had applied unscientific methods. Initially, their arguments remained unheard but around 1990, the German government reconsidered its viewpoint. After setting the political threshold of particulate emissions to 0.08 g/km, diesel cars once again received the label “particularly low-emission” as well as a favorable tax treatment. This particulate standard was insofar a political threshold, as most German-produced diesel cars easily passed the limit. Consequently, the German government did not apply the precautionary principle but rather—like the US government before—favored economic interest over health effects, despite the proven carcinogenic effect of diesel particulate emissions.

Second, as engineers failed to provide a reliable and durable particulate filter—the most promising pollution control technology—by the mid-1980s, the consequences for the diesel car’s reputations were severe. The public soon stigmatized diesel cars as sluggish, unreliable, and “cancer-causing”. Consequently, consumer interest in diesel cars vanished. Yet, this also had an impact on the intellectual context as it redefined the engineering solutions on how to “clean” diesel emissions. After failing with the filter, engineers at Mercedes-Benz and Volkswagen tried to lower particulate emissions by improving the internal combustion of the diesel engine at the end of the 1980s. From an engineer’s viewpoint, this appeared a logical step since it allowed lowering the emitted mass of particulates; and environmental regulation measured the amount of particulate emissions in grams emitted per kilometer or mile. Latest scientific research indicated at that time, however, that the engineering solution was ineffective. Toxicologists noted that not the mass of emitted diesel particulate defined the cancer risk but rather the number of emitted particles. Yet, an improved internal combustion did not affect the latter. Quite the contrary, lowering the mass of particulates, they became smaller and thus could more easily penetrate the lungs. Scientists thus argued that the engineering solutions increased the cancer risk. Yet, this viewpoint remained unheard in the political debates of the early 1990s. In particular, government officials lent an ear to scientists, engineers, and managers of the automotive industry that presented a divergent interpretation of the research results: they categorically denied the cancer risks of diesel particulate emissions. Hence, and as shown above, diesel cars were not banned in Germany.

US and German regulatory policies also varied, indicating another important aspect of intellectual differences. US regulatory policy relied mostly on mathematical risk assessment and management as well as on epidemiological data that had to withstand in public and often controversial hearings. The German government, however, tended to base its line of argumentation—in addition to epidemiology—on the results of animal testing. Furthermore, the debate on the carcinogenic effects of diesel particulates was public; but the political negotiations took place behind closed doors, giving more leverage to economic interests. This can be considered a peculiarity of the German (or European approach) up to the 1990s.

Third, the geographical context mattered. While neither the Carter nor the Reagan Administration followed the precautionary principle in its regulatory policy as shown in the literature (Langston 2010; Vogel 2012), the State of California diverged. Its Air Resources Board (CARB) aimed to reduce drastically the emission of cancer-causing chemicals. In particular, the geographical context mattered because Los Angeles County suffered from severe air pollution. While automakers did not have any major plants in California, the state was the most important internal market within the USA. Moreover, its environmental regulation served as a role model for several other states within the USA that adopted California’s emission standards such as New York.

The argument presented showed that between the 1970s and 2010s automakers and their lobbying groups as well as researchers had “flexibly” interpreted scientific research results. This is a crucial difference to the Volkswagen diesel scandal of the 2010s because a car manufacturer had openly broken the law. This did not happen in the period reviewed. Moreover, German carmakers knew what was at stake economically, because they wanted to conquer the North American market with their “clean” diesel technology—and its promise of tremendous profits. This end justified the means to break both legal and ethical barriers by cheating on emission tests but also conducting inhalation experiments with both monkeys and humans. Probably the latter should provide irrefutable scientific evidence on the non-toxicity of diesel exhaust—an assessment that could not be inferred from animal tests as the automotive industry has always been keen to emphasize. It also shows how the diverging acceptance of diesel cars in the US and in West Germany is linked to the reputation of diesel cars and their emissions control technologies; the scientific assessment of diesel exhaust fumes; and to the political regulation of diesel vehicles.

## Acknowledgements

I would like to thank the anonymous referees and the editors as well as Jennifer L. Rodgers, Rüdiger Graf, and Andreas Weiß for their comments and suggestions that substantially improved the manuscript.

## Archival Sources

- Bundesarchiv Koblenz B 295/27826.
- Jimmy Carter Library JC-AINFL: Series Ron B. Lewis’ Subject Files, 1977–1981, Box 78.
- Jimmy Carter Library JC-AINFL: Series Ron B. Lewis’ Subject Files, 1977–1981, Box 79.
- Jimmy Carter Library JC-CEA: Series Charles L. Schultze’s Subject Files, 1977–1981, Box 21.
- Jimmy Carter Library JC-DPS: Series Kathryne Bernick Files, 1979–1980, Box 9.
- Jimmy Carter Library JC-DPS: Series Richard Neustadt Files, 1976–1980, Box 70.

## Footnotes

1 See Eckl-Dorna (2018); Doll & Vetter (2018); *Spiegel Online* (2018); Brand et al. (2016); Grill et al. (2018); Ewing (2018a; 2018b).

2 See National Cancer Act of 1971. URL: https://dtp.cancer.gov/timeline/noflash/milestones/M4_Nixon.htm (14.02.2020).

3 See Ethridge (1978: 50); National Research Council (1981: 16, 22–3); Pott & Heinrich (1988: 687); Heinrich 1992: 67. For the general controversies on chemicals and their impacts on humans as well as the role of EPA, see Jasanoff (1992: 201; 1990: 20–38).

4 See Jimmy Carter Library JC-AINFL: Series Ron B. Lewis’ Subject Files, 1977–1981, Box 79, Diesel-Environmental, Protection Agency (EPA), 11/79–2/80; letter Michael P. Walsh to Larry Linden, Washington, D.C., 10 January 1980; Jimmy Carter Library JC-DPS: Series Richard Neustadt Files, 1976–1980, Box 70, “Regulatory Reform—Diesel Particulates”, Diesels-Environmental Protection Agency (EPA), 11/79–2/80; memorandum Larry White to Bill Nordhaus and Frank Press, “Automotive Diesel Particulate Emissions,” 25 October 1978, p. 1.

5 See Kurani & Sperling (1988: 23).

6 See McGonegal (1979: 28).

7 Knepper (1980: 83).

8 See Jimmy Carter Library JC-CEA: Series Charles L. Schultze’s Subject Files, 1977–1981, Box 21 Diesel Particulates, Memorandum Larry White to Bill Nordhaus, 4 December 1978.

9 National Research Council (1981*: *XI).

10 See Davis & Diegel (2004 table 4.5); National Research Council (1982a: XI); National Research Council (1981: XI); National Research Council (1982b).

11 See National Research Council (1982a: XII–XVII, 27); Jimmy Carter Library JC-DPS: Series Kathryne Bernick Files, 1979–1980, Box 9, Murphy (Thomas A., Chairman of Gen’l Motors)—Diesel/NOX (Oxides of Nitrogen) (Waivers) Memorandum Phil Smith to Larry Linden and Si Lazarus, Washington, D.C., 19 November 1979; Jimmy Carter Library JC-AINFL: Series Ron B. Lewis’ Subject Files, 1977–1981, Box 78, Autos 11/79–2/80, Memorandum Kitty Bernick to Ron Lewis et al., Washington, D.C., 22 January 1980. For a summary on the political debate, see (Neumaier 2014: 438–439, 434–444; 2010b: 148–150, 157).

12 See National Research Council (1982a: XIII, 29).

13 National Research Council (1982a: 29).

14 See National Research Council (1982a: 29).

15 See SAE Automotive Engineering (1981: 79, 84–85; 1984: 87–91); Schnabel (1986: 138); National Research Council (1982a).

16 See SAE Automotive Engineering (1981: 80–88); Berg (1982: 296).

17 SAE Automotive Engineering (1981: 89).

18 SAE Automotive Engineering (1981: 89).

19 See SAE Automotive Engineering (1981: 79–89).

20 See Berg (1982: 297–298).

21 National Research Council (1982a: 29).

22 See Pasztor (1982: 23).

23 See SAE Automotive Engineering (1983: 30).

24 Ward’s Reports (1982: 67; 1987: 93, 186), Consumer Reports (1980: 394).

25 See Kimball (1984: 128–129).

26 Ward’s Communication (1986:171; 1987: 186).

27 See Schuon (1984: D2); Ward’s Reports: (1982–1986); California Air Resources Board (1989: 2); Berg (1996: 125–126); Kurani & Sperling (1988: 23, 25).

28 See *Los Angeles Times* (1984: I11); *The New York Times* (1986: A17).

29 See *Los Angeles Times* (1987: 2); *The New York Times* (1987: A29); von Bredow et al. (2005: 86).

30 See *Road & Track* (1980); Schroeder (1992, 1996).

31 See Phillips III (1990: 131); Markus (2004).

32 For a summary of the public discourse see Neumaier (2014: 441–443).

33 Letter, Philip Handler to Douglas M. Costle et al., 30 September 1980, in: National Research Council (1982b).

34 Oppenheimer et al*.* (2019: 16).

35 National Research Council (1981: 1).

36 National Research Council (1981: V–VI, XII, 1–2).

37 National Research Council (1981: 2).

38 National Research Council (1981: 4).

39 National Research Council (1981: 32).

40 See National Research Council (1981: 2–3, 32–35, 54, 62, 69); Heinrich (1992: 67).

41 National Research Council (1981: 2).

42 See National Research Council (1981: 5, 7, 54, 62, 69, 87–89, 93–94); Pott & Heinrich (1988: 686–687); Klingenberg et al. (1991: 121–122).

43 National Research Council (1981: 57).

44 Vostal (1980: 916).

45 Vostal (1980: 924).

46 See Vostal (1980: 931).

47 See National Research Council (1981: 55–60); United States (1981); Vostal (1980: 916, 919, 922–25, 929). For a summary of Vostal’s arguments, see Neumaier (2014: 442–443; 2010b: 155).

48 National Research Council (1981: 3).

49 See National Research Council (1982a: XVI–XVIII).

50 See Heinrich (1992: 67). For a controversy on the quantitative risk assessment of ethylene dibromide (EDB) in the first half of the 1980s see Jasanoff (1990: 135–137).

51 Cumming (1983: 81).

52 See Harris (1981; 1983a); Cumming (1983: 81–82). For the quote see: Cumming (1983: 82). For a summary on research on risks see Zachmann (2014: 5, 27–28), Mohun (2013).

53 Harris (1983a: 83–84).

54 Harris (1983a: 91).

55 See National Research Council (1981: 6, 95, 126–138); National Research Council (1982a: 71–72, 111–112); Harris (1983a: 91, 97).

56 Cuddihy & McClellan (1983: 124).

57 See Harris (1983a: 91, 96); Albert et al. (1983: 101–117); Cuddihy & McClellan (1983); Houser et al. (1983: 125–128); Schwing et al. (1983: 129–131); Harris (1983b); Travis & Munro (1983: 147–155). The Lovelace Respiratory Research Institute became infamous, when it exposed monkeys to the emissions of a Volkswagen diesel equipped with a “defeat device”. (Ewing 2018a).

58 See National Research Council (1982a: 112).

59 See Martin (2004); Sullivan (2004); Pasztor (1981).

60 *The New York Times* (1982: B17). For a critical analysis of the EPA during and after Gorsuch’s time as administrator see Jasanoff (1992: 198–199).

61 Oppenheimer et al. (2019: 16).

62 See Kurani & Sperling (1988: 24).

63 *Auto Motor und Sport* (1992: 41).

64 See Carb-Chefin Mary Nichols: VW-Abgasbetrug vermutlich kein Einzelfall. URL: https://www.bundestag.de/dokumente/textarchiv/2017/kw10-pa-ua-abgas-495176 (19.06.2020).

65 See Kraftfahrt-Bundesamt (1995: 36); *ADAC Motorwelt* (1984; 1985); Goblirsch (1986: 44–50); *Auto Motor und Sport* (1987c: 6); *Auto Motor und Sport* (1987a: 59).

66 See Goblirsch (1985); Westheide (1987: 51–80).

67 See Vorwort, in: Kommission Reinhaltung der Luft im VDI und DIN (1991).

68 Sauer (1987: 65).

69 See Sauer (1987); *ADAC Motorwelt* (1984: 28); Droste (1984: 86–90); Goblirsch (1986). For a summary of the debate see Neumaier (2014: 446–449; 2010b: 175–180).

70 See Goblirsch (1986); *Der Spiegel* (1986a: 120); Heinrich (1991: 1–2, 5); Pott (1991: 215, 222–223); Kraftfahrt-Bundesamt (1995: 36).

71 See *Der Spiegel* (1986a); *Auto Motor und Sport* (1987b).

72 See Verband der Automobilindustrie (1988: 5–7).

73 Oreskes & Conway (2010: 136).

74 See Verband der Automobilindustrie (1988: 10–11).

75 See Verband der Automobilindustrie (1988: 10–11, 18); *Der Spiegel *(1986b: 19–21). This assessment resembled the EPA cancer risk assessment of diesel particulates published eight years earlier. In addition, the VDA also stressed the validity of its statement by quoting the UBA statement from 1986/87 that had negated the connection between diesel exhaust and cancer, omitting to mention how this press release had originated. From this viewpoint, the diesel car could receive both labels: “eco-friendly” and “safe enough” to public health. (Neumaier 2020: 96–100). For a general summary on the controversies, see Neumaier (2010b: 176–182, 2014: 449–450).

76 See Heinrich (1991: 3–4, 6); *Der Spiegel* (1989: 202).

77 See *Der Spiegel* (1989: 201–202); Pott (1991: 214, 223, 226); Pott & Heinrich (1988: 687); Wichmann & Büske-Hohlfeld (1991).

78 See *Der Spiegel* (1989: 201–202).

79 See Pott & Heinrich (1988: 686); Pott (1991: 211, 223–225); Heinrich (1991: 6–7).

80 See Pott (1991: 212, 227–229).

81 *Der Spiegel* (1989: 202).

82 ADAC Motorwelt (1989a: 32).

83 See *Auto Motor und Sport* (1986).

84 *Der Spiegel* (1989: 201). Similar viewpoint took the head of engine development of Mercedes-Benz Kurt Obländer. (Obländer: 1991: 245).

85 See *Der Spiegel* (1989: 201–202); *Motortechnische Zeitschrift* (1989: 398); Hack (1989: 104–107); Fortnagel & Moser (1992: 14–20); König (1990: 34–38); Klingenberg et al. (1991: 128); Obländer (1991: 245–247); Haschek (1991: 223); Sauer (1990: 22); Heinrich (1991: 1, 4); von Bredow et al. (2005: 86); California Air Resources Board (1989: 2).

86 See Obländer (1991: 247); Klingenberg et al. (1991: 121, 127).

87 See Pott & Heinrich (1988: 686–687); Pott (1991: 211–212, 215–215, 231); Heinrich (1992: 65–66, 68, 70–71); Heinrich (1991: 5, 8–9).

88 See Heinrich (1991: 1, 9); Heinrich (1992: 71); Pott & Heinrich (1988: 687–668); Pott (1991: 214–218).

89 See Pott (1991: 211–212, 230).

90 See Winterhagen: (1993: 489).

91 See Röthig (1989: 36); Linke (1989: 53); Winterhagen (1993: 489); Bundesarchiv Koblenz B 295/27826, Blümel, Herrmann u. a. 1992. *Erster Zwischenbericht des Umweltbundesamtes zum Rußfilter-Großversuch*, Berlin: 9–10.

92 Health Effects Institute (1995: 5).

93 See Health Effects Institute (1995: 5–6); *Auto Motor und Sport* (1995: 3). For further details see Neumaier (2010b: 195–196).

94 See *ADAC Motorwelt* (1989b: 3); *ADAC Motorwelt *(1989c: 50–53); Goblirsch (1991).

## References

[CR1] ADAC Motorwelt 1984. Schwarzer Peter oder Saubermann? *ADAC Motorwelt* (6):28.

[CR2] ADAC Motorwelt 1985. Jeder fünfte ein Nagler. *ADAC Motorwelt* (11): 8.

[CR3] ADAC Motorwelt 1989a. Krebs durch Diesel? *ADAC Motorwelt* (1): 32.

[CR4] ADAC Motorwelt 1989b. Treibhaus. *ADAC Motorwelt* (10): 3.

[CR5] ADAC Motorwelt 1989c. Wieviel Schuld hat das Auto? *ADAC Motorwelt* (10): 50–53.

[CR27] Albert RE, Lewtas J, Nesnow S, Thorslund TW, Anderson E (1983). Comparative potency method for cancer risk assessment: application to diesel particulate emissions. Risk Analysis.

[CR6] Auto Motor und Sport 1986. Hätten Sie’s gewußt, Herr Diesel? *Auto Motor und Sport *(12): 26–32.

[CR7] Auto Motor und Sport 1987a. Unendliche Geschichte. *Auto Motor und Sport* (10): 59.

[CR8] Auto Motor und Sport 1987b. Ich bin ein bekennender Techniker. *Auto Motor und Sport* (14): 168–169.

[CR9] Auto Motor und Sport 1987c. Feilschen um EG-Diesel. *Auto Motor und Sport* (16): 6.

[CR10] Auto Motor und Sport 1992. Die Zukunft des Diesel: Filterfrisch? *Auto Motor und Sport* (5): 41.

[CR11] Auto Motor und Sport 1995. Mit schönem Ruß. *Auto Motor und Sport* (24): 3.

[CR28] Berg W (1982). Aufwand und Probleme für Gesetzgeber und Automobilindustrie bei der Kontrolle der Schadstoffemissionen von Personenkraftwagen mit Otto- und Diesel-Motoren dargestellt am Beispiel ausgewählter Exportländer.

[CR29] Berg W, Gruden D (1996). Weltweite Abgas-Gesetzgebung für PKW: Was erwartet die Automobil-Industrie?. Die ökologische Dimension des Automobils.

[CR30] Borg KL (2014). Introduction: constructing sociotechnical environments—aurality, air quality, and automobiles. Technology & Culture.

[CR31] Brand P, Bertram J, Chaker A, Jörres RA, Kronseder A, Kraus T, Gube M (2016). Biological effects of inhaled nitrogen dioxide in healthy human subjects. International Archives of Occupational and Environmental Health.

[CR32] Bredow, Rafaela von, Dominik Cziesche, Veronika Hackenbroch, Dietmar Hawranek, Sebastian Knauer, Michael Sontheimer, Andreas Wassermann and Christian Wüst 2005. Die unsichtbare Gefahr. *Der Spiegel* (14): 78–94.

[CR33] Brickman R, Jasanoff S, Ilgen T (1985). Controlling chemicals. The politics of regulation in europe and the United States.

[CR34] California Air Resources Board (1989). Progress report on reducing exposure to diesel engine emissions. Response to senate concurrent resolution no. 100.

[CR12] Consumer Reports 1980. Are Diesels Durable? CU Learns the Hard Way. *Consumer Reports* (45): 394.

[CR35] Cuddihy RG, McClellan RO (1983). Evaluating lung cancer risks from exposures to diesel engine exhaust. Risk Analysis.

[CR36] Cumming RB (1983). The projection of long-term technological trends and their associated risks. Risk Analysis.

[CR37] Davis SC, Diegel SW (2004). Transportation energy data book.

[CR13] Der Spiegel 1986a. Ruß mit Rabatt*. Der Spiegel* (48): 120–122.

[CR14] Der Spiegel 1986b. Diesel: Krebs aus dem Auspuff? *Der Spiegel* (52): 19–21.

[CR15] Der Spiegel 1989. Druck von außen*. Der Spiegel* (6): 201–202.

[CR38] Doll, Nikolaus and Philipp Vetter 2018. *Der große Irrtum vom Ende des Dieselskandals*. URL: https://www.welt.de/wirtschaft/article172940396/Volkswagen-Der-grosse-Irrtum-vom-Ende-des-Dieselskandals.html (17.06.2020).

[CR39] Droste, Michael 1984. Diesel im Fegefeuer. *Auto Zeitung* (26): 86–90.

[CR40] Eckl-Dorna, Wilfried 2018. *Wie Forscher jahrelang halfen, den Diesel-Betrug zu vertuschen. Tierversuche bei Volkswagen*. URL: https://www.spiegel.de/wirtschaft/unternehmen/vw-daimler-bmw-was-hinter-der-forschungsinitiative-eugt-steckt-a-1190445.html (17.06.2020).

[CR41] Ethridge, John 1978. Diesel Report. *Motor Trend,* November 1987: 50–51.

[CR42] Ewing, Jack 2018a. *10 Monkeys and a Beetle: Inside VW’s Campaign for ‘Clean Diesel*’. URL: https://www.nytimes.com/2018/01/25/world/europe/volkswagen-diesel-emissions-monkeys.html (17.06.2020).

[CR43] Ewing, Jack. 2018b. *How I Uncovered Volkswagen’s Rigged Monkey Experiments*. URL: https://www.nytimes.com/2018/02/22/insider/volkswagen-monkey-experiments.html (17.06.2020).

[CR44] Fortnagel M, Moser P (1992). Die Mercedes-Benz Dieselmotorbaureihe für Personenkraftwagen mit Abgasrückführung und Oxidationskatalysator. Motortechnische Zeitschrift.

[CR47] Goblirsch R (1991). Rentiert sich jetzt der Diesel?. ADAC Motorwelt.

[CR45] Goblirsch, Ruth 1985.Der neue Fahrplan für das umweltfreundliche Auto. *ADAC Motorwelt* (5): 26–29.

[CR46] Goblirsch, Ruth 1986. Jetzt ist der Diesel fällig. *ADAC Motorwelt* (9): 44–50.

[CR48] Grill, Markus, Max Hägler and Klaus Ott 2018. *Autobauer benutzten Wissenschaftler, um Gefahren durch Diesel zu verharmlosen*. URL: sueddeutsche.de. https://www.sueddeutsche.de/wirtschaft/schadstofftests-autobauer-benutzten-wissenschaftler-um-gefahren-durch-diesel-zu-verharmlosen-1.3845136 (17.06 2020).

[CR49] Hack, Gert 1989. Reine Postsache. *Auto Motor und Sport* (10): 104–107.

[CR50] Harris JE (1981). Potential risk of lung cancer from diesel engine emissions.

[CR51] Harris JE (1983). Diesel emissions and lung cancer. Risk Analysis.

[CR52] Harris JE (1983). Diesel emissions and lung cancer revisited. Risk Analysis.

[CR53] Haschek, Brigitte 1991. Nagelprobe. *Auto Motor und Sport* (6): 222–225.

[CR55] Health Effects Institute (1995). Diesel Exhaust: A Critical Analysis of Emissions, Exposure, and Health Effects: A Special Report of the Institute’s Diesel Working Group..

[CR56] Heinrich U, VDI-Gesellschaft Fahrzeugtechnik (1991). Gesundheitliche Wirkung der Dieselabgasemission: Stand der Forschung. Abgas- und Geräuschemissionen von Nutzfahrzeugen.

[CR57] Heinrich U, Kommission Reinhaltung der Luft im VDI und DIN (1992). Das kanzerogene Potential von Luftschadstoffen – Die Bedeutung von Kfz-Abgasen. Umweltschutz in Städten: Emissionsminderung – Entsorgung – Energie – Planung. Tagung.

[CR58] Houser HB, Mortimer EA, Haimes YY, Rosenkranz HS (1983). Diesel Emissions, Short-Term Bioassays, and Lung Cancer. Risk Analysis.

[CR59] Jasanoff S (1986). Risk management and political culture. A comparative study of science in the policy context.

[CR60] Jasanoff S (1990). The fifth branch. Science advisers as policymakers.

[CR61] Jasanoff S (1992). Science, Politics, and the Renegotiation of the Expertise at EPA. Osiris.

[CR62] Jasanoff S (2005). Designs on nature. Science and democracy in Europe and the United States.

[CR63] Kimball, Steve 1984. Braking for the Bad Times. *Road & Track,* November 1984: 126–134.

[CR64] Klingenberg H, Schürmann D, Lies K-H, Kommission Reinhaltung der Luft im VDI und DIN (1991). Dieselmotorabgas – Entstehung und Messung. Krebserzeugende Stoffe in der Umwelt: Herkunft, Messung, Risiko, Minimierung: Kolloquium.

[CR65] Knepper, Mike 1980. Mercedes-Benz 300 TD. *Car and Driver,* March 1980: 83–87.

[CR66] Kommission Reinhaltung der Luft im VDI und DIN (1991). Krebserzeugende Stoffe in der Umwelt: Herkunft, Messung, Risiko, Minimierung.

[CR67] König, Wolfgang 1990. Sauberer geht’s nicht. *Auto Motor und Sport* (20): 34–38.

[CR68] Kraftfahrt-Bundesamt (1995). Statistische Mitteilungen des Kraftfahrt-Bundesamtes.

[CR69] Kurani KS, Sperling D (1988). Rise and Fall of Diesel Cars: A Consumer Choice Analysis. Transportation Research Record.

[CR70] Langston N (2010). Toxic bodies. Hormone disruptors and the legacy of DES.

[CR71] Linke H (1989). Abgastrübung bald von untergeordneter Bedeutung?. Automobil Revue.

[CR16] Los Angeles Times 1984. Mercedes-Benz Advertisement. *Los Angeles Times,* 20 December 1984: I10–I11.

[CR17] Los Angeles Times 1987. 9.000 Diesel Models Recalled by Mercedes. *Los Angeles Times,* 18 June 1987: 2.

[CR72] Markus, Frank 2004. Diesels now offer superior performance, fuel economy, and longevity, but have CARB and the EPA made outlaws of them?’ *Car and Driver*, March 2004: 118–122.

[CR73] Martin, Douglas 2004. Anne Gorsuch Burford, 62, Reagan E.P.A. Chief, Dies. *The New York Times,* 22 July 2004: C13.

[CR75] McCarthy T (2007). Auto mania. Cars, consumers, and the environment.

[CR76] McGonegal, Ro 1979. Dieselmania! *Motor Trend*, August 1979: 28–42.

[CR77] Mohun AP (2013). Risk: negotiating safety in American Society.

[CR18] Motortechnische Zeitschrift (1989). VW-Umwelt-Dieselmotor mit geringer Rauchentwicklung und verminderten Kohlenwasserstoffen. Motortechnische Zeitschrift.

[CR78] National Research Council (1981). Health effects of exposure to diesel exhaust: the report of the health effects panel of the diesel impacts study committee.

[CR79] National Research Council (1982). Diesel cars: benefits, risks, and public policy: final report of the diesel impacts study committee, assembly of engineering.

[CR80] National Research Council (1982). Diesel technology: report of the technology panel of the diesel impacts study committee.

[CR81] Neumaier C (2010). Design Parallels, Differences and … a Disaster. American and German Diesel Cars in Comparison, 1968–1985. ICON. Journal of the International Committee for the History of Technology.

[CR82] Neumaier C (2010). Dieselautos in Deutschland und den USA: Zum Verhältnis von Technologie, Konsum und Politik, 1949–2005.

[CR83] Neumaier C (2010). Von kulturellen Präferenzen und technologischen Fehlschlägen. Der Diesel-Pkw im transatlantischen Vergleich Deutschland – USA, 1976–1985. Technikgeschichte.

[CR84] Neumaier C (2014). Eco-Friendly vs. Cancer-Causing: Perceptions of Diesel Cars in West Germany and the United States, 1970–1990. Technology & Culture.

[CR85] Neumaier C, Heßler M (2020). Rechnen mit Emotionen. Kontroversen um die Gesundheitsrisiken von Dieselabgasen in Deutschland und den USA in den 1980er Jahren. Technikemotionen.

[CR86] Obländer K, Kommission Reinhaltung der Luft im VDI und DIN (1991). Möglichkeiten der Schadstoff-Minimierung bei Dieselmotoren. Krebserzeugende Stoffe in der Umwelt: Herkunft, Messung, Risiko, Minimierung. Kolloquium.

[CR87] Oppenheimer M, Oreskes N, Jamieson D, Brysse K, O’Reilly J, Shindell M, Wazeck M (2019). Discerning experts. The practices of scientific assessment for environmental policy.

[CR88] Oreskes N, Conway EM (2010). Merchants of doubt: how a handful of scientists obscured the truth on issues from tobacco smoke to global warming.

[CR89] Pasztor, Andy 1981. Studies That Find Diesel Fumes Benign Encourage the Easing of Engine Controls. *The Wall Street Journal,* 30 July 1981: 13.

[CR90] Pasztor, Andy 1982. Battle Brewing Over Exhaust of Diesel Cars. *The Wall Street Journal,* 14 June 1982: 23.

[CR91] Phillips J (1990). Mercedes-Benz 300 D 2.5 Turbo. Car and Driver.

[CR92] Porter TM (1995). Trust in numbers. The pursuit of objectivity in science and public life.

[CR93] Pott F, Kommission Reinhaltung der Luft im VDI und DIN (1991). Dieselmotorabgas – Tierexperimentelle Ergebnisse zur Risikoabschätzung. Krebserzeugende Stoffe in der Umwelt: Herkunft, Messung, Risiko, Minimierung. Kolloquium.

[CR94] Pott F, Heinrich U (1988). Neue Erkenntnisse über die krebserzeugende Wirkung von Dieselmotorabgas. Zeitschrift für die gesamte Hygiene und ihre Grenzgebiete.

[CR95] Proctor RN (1995). Cancer wars: how politics shapes what we know and don’t know about cancer.

[CR19] Road & Track 1980. Volkswagen Rabbit Diesel. *Road & Track,* November 1980: 60–62.

[CR96] Röthig G (1989). Stinker oder Saubermann?. Auto Zeitung.

[CR20] SAE Automotive Engineering (1981). Particulate filters: a ‘must’ for light-duty diesels?. SAE Automotive Engineering.

[CR21] SAE Automotive Engineering (1983). Diesel particulate filters: an update. SAE Automotive Engineering.

[CR22] SAE Automotive Engineering (1984). Diesel particulate traps: three approaches. SAE Automotive Engineering.

[CR97] Sauer, Heinrich 1987. Ohne Gewähr. *Auto Motor und Sport* (10): 60–70.

[CR98] Sauer, Heinrich 1990. Sauberer Zauber*. Auto Motor und Sport* (25): 22.

[CR99] Schnabel W (1986). Rußfilter für den Serieneinbau bei Dieselmotoren. Motortechnische Zeitschrift.

[CR100] Schroeder, Don 1992. Volkswagen Jetta ECOdiesel, *Car and Driver,* November 1992: 150–151.

[CR101] Schroeder, Don 1996. Volkswagen Passat TDI Diesel. *Car and Driver,* May 1996: 125–129.

[CR102] Schuon, Marshall 1984. Cutting Down on Diesel’s Ills. *The New York Times,* 15 November 1984: D2.

[CR103] Schwing RC, Evans L, Schreck RM (1983). Uncertainties in diesel engine health effects. Risk Analysis.

[CR23] Spiegel Online 2018: “*Ethisch in keiner Weise zu rechtfertigen*”*. Reaktionen auf Abgasversuche*. URL: https://www.spiegel.de/wirtschaft/unternehmen/vw-aufsichtsratschef-verurteilt-abgasversuche-an-menschen-und-affen-a-1190347.html (17.06.2020).

[CR105] Sullivan, Patricia 2004. Anne Gorsuch Burford, 62, Dies; Reagan EPA Director. *The Washington Post,* 22 June 2004: B6.

[CR24] The New York Times 1982. California Plans Fight on Carcinogens in Air. *The New York Times,* 30 September 1982: B17.

[CR25] The New York Times 1986. Mercedes-Benz Advertisement. *The New York Times*, 15 July 1986: A16–A17.

[CR26] The New York Times 1987. Mercedes Recalls 9,000. *The New York Times,* 18 June 1987: A29.

[CR106] Travis CC, Munro NB (1983). Potential health effects of light-duty diesel exhaust. Risk Analysis.

[CR107] United States. Congress. House. Committee on Science and Technology. Subcommittee on Natural Resources and Environment, EPA Diesel Particulate Standards 1981. *Hearings Before the Subcommittee on Natural Resources and Environment of the Committee on Science and Technology*, Washington, D.C.: U.S. House of Representatives, Ninety-Sixth Congress, Second Session, October 1 & 2, 1980, No. 181.

[CR108] Verband der Automobilindustrie (1988). Dieselabgase – eine Gefahr für den Menschen?.

[CR109] Vogel D (2012). The politics of precaution: regulating health, safety, and environmental risks in europe and the United States.

[CR110] Vostal JJ (1980). Health aspects of diesel exhaust particulate emissions. Bulletin of the New York Academy of Medicine.

[CR111] Ward’s Report (1982). 1982 Ward’s Automotive Yearbook: Forty-Fourth Volume.

[CR112] Ward’s Report (1983). 1983 Ward’s Automotive Yearbook: Forty-Fifth Volume.

[CR113] Ward’s Report (1984). 1984 Ward’s Automotive Yearbook: Forty-Sixth Volume.

[CR114] Ward’s Reports (1985). 1985 Ward’s Automotive Yearbook: Forty-Seventh Volume.

[CR115] Ward’s Reports (1986). Ward’s automotive yearbook.

[CR116] Ward’s Reports (1987). Ward’s automotive yearbook.

[CR117] Weingart P (2005). Die Stunde der Wahrheit? Zum Verhältnis der Wissenschaft zu Politik, Wirtschaft und Medien in der Wissensgesellschaft.

[CR118] Westheide, Eberhard 1987. *Die Einführung bleifreien Benzins und schadstoffarmer Pkw in der Bundesrepublik Deutschland mit Hilfe ökonomischer Anreize. Umweltpolitische Effizienz sowie wettbewerbs- und außenhandelspolitische Implikationen der Umstellung der Märkte für Benzin und Personenkraftwagen. *(Luftreinhaltung in Forschung und Praxis, 4). Berlin: Erich Schmidt Verlag.

[CR119] Wichmann H-E, Büske-Hohlfeld I, Kommission Reinhaltung der Luft im VDI und DIN (1991). Epidemiologische Befunde zum Krebsrisiko durch Dieselmotorabgase. Krebserzeugende Stoffe in der Umwelt. Herkunft, Messung, Risiko, Minimierung. Kolloquium.

[CR120] Winterhagen J (1993). Die Kanzerogenität von Rußpartikeln im Dieselabgas. Motortechnische Zeitschrift.

[CR121] Zachmann K, Klüppelberg C, Straub D, Welpe IM (2014). Risk in historical perspective: concepts, contexts, and conjunctions. Risk—A multidisciplinary introduction.

